# Functional Characterization of a Syntaxin Involved in Tomato (*Solanum lycopersicum*) Resistance against Powdery Mildew

**DOI:** 10.3389/fpls.2017.01573

**Published:** 2017-09-20

**Authors:** Valentina Bracuto, Michela Appiano, Zheng Zheng, Anne-Marie A. Wolters, Zhe Yan, Luigi Ricciardi, Richard G. F. Visser, Stefano Pavan, Yuling Bai

**Affiliations:** ^1^Section of Genetics and Plant Breeding, Department of Plant, Soil and Food Science, University of Bari Aldo Moro Bari, Italy; ^2^Plant Breeding, Wageningen University & Research Wageningen, Netherlands; ^3^Institute of Vegetables and Flowers, Chinese Academy of Agricultural Sciences Beijing, China

**Keywords:** *mlo* resistance, non-host resistance, *Blumeria graminis* f. sp. *hordei*, *Oidium neolycopersici*, tomato syntaxins

## Abstract

Specific syntaxins, such as Arabidopsis AtPEN1 and its barley ortholog ROR2, play a major role in plant defense against powdery mildews. Indeed, the impairment of these genes results in increased fungal penetration in both host and non-host interactions. In this study, a genome-wide survey allowed the identification of 21 tomato syntaxins. Two of them, named *SlPEN1a* and *SlPEN1b*, are closely related to *AtPEN1*. RNAi-based silencing of *SlPEN1a* in a tomato line carrying a loss-of-function mutation of the susceptibility gene *SlMLO1* led to compromised resistance toward the tomato powdery mildew fungus *Oidium neolycopersici*. Moreover, it resulted in a significant increase in the penetration rate of the non-adapted powdery mildew fungus *Blumeria graminis* f. sp. *hordei*. Codon-based evolutionary analysis and multiple alignments allowed the detection of amino acid residues that are under purifying selection and are specifically conserved in syntaxins involved in plant-powdery mildew interactions. Our findings provide both insights on the evolution of syntaxins and information about their function which is of interest for future studies on plant–pathogen interactions and tomato breeding.

## Introduction

In eukaryotic cells, compartmentalization through the endomembrane apparatus and exocytosis require a highly regulated transport system. Soluble *N*-ethylmaleimide-sensitive-factor attachment protein receptor (SNARE) proteins are involved in such a transport, as they mediate the fusion of membranes of cargo-containing small shuttles, referred to as vesicles, and target membranes (Lipka et al., 2007). According to their localization, SNARE proteins can be classified into vesicle-associated (v-SNAREs) and target-membrane-associated (t-SNAREs) (Söllner et al., 1993). Alternatively, they can be classified into Q-SNARE and R-SNARE proteins, which contain either arginine or glutamine at the center of the SNARE domain, respectively (Fasshauer et al., 1998). Typically, SNARE complexes involved in membrane fusion are tetrameric coiled-coil structures containing one R-SNARE protein anchored to the vesicle and one of each of the three Q-SNARE proteins, namely Qa- (also referred to as syntaxins or SYPs), Qb- and Qc-SNAREs (Fasshauer et al., 1998; Bock et al., 2001; Schilde et al., 2008). Based on sequence homology, syntaxins of the Qa SNARE family can be divided into five subfamilies known as SYP-1, -2, -3, -4, -8 (Sanderfoot et al., 2000; Bock et al., 2001).

Ascomycete fungi of the order of Erysiphales cause the powdery mildew disease on 1000s of plant species and lead to massive economic losses in agricultural settings (Takamatsu, 2004). Specific members of the syntaxin protein family, such as Arabidopsis AtPEN1, barley ROR2, and grapevine VvPEN1, are essential components of a SNARE-dependent antimicrobial secretion pathway that limits the penetration of powdery mildew fungi (Collins et al., 2003; Feechan et al., 2013). In compatible interactions between barley and the barley powdery mildew pathogen *Blumeria graminis* f. sp. *hordei* (*Bgh*), *ror2* mutations lead to a significant increase of fungal entry, thus indicating a functional role in basal defense mechanisms (Collins et al., 2003). In non-host interactions, involving Arabidopsis and barley with non-adapted powdery mildew species, *Atpen1* and *ror2* mutations also result in increased penetration rates (Peterhänsel et al., 1997; Collins et al., 2003; Assaad et al., 2004). However, further fungal growth is prevented by the occurrence of cell hypersensitive response, which is assumed to act as a post-invasion defense mechanism (Collins et al., 2003; Trujillo et al., 2004; Lipka et al., 2005).

Syntaxin-dependent exocytosis is also required for *mlo*-based defense, exhibited by plants harboring loss-of-function mutations of *MLO* susceptibility genes and associated with the failure of fungal penetration in correspondence of thick cell wall appositions termed papillae (Pavan et al., 2010; Seifi et al., 2014). In barley, *ror2* mutations break *mlo* resistance to *Bgh*, resulting in the restoration of visible disease symptoms (Freialdenhoven et al., 1996; Collins et al., 2003). In Arabidopsis, *Atmlo2/Atpen1* double mutants display higher penetration rate of the fungal species *Golovinomyces cichoracearum* than single *Atmlo2* mutants. However, they do not exhibit increased fungal sporulation and still appear phenotypically resistant (Consonni et al., 2006). The *mlo*-based defense and syntaxins also operate at the non-host level. Indeed, barley *mlo* mutants show enhanced penetration resistance to the non-adapted wheat powdery mildew fungus *Blumeria graminis* f. sp. *tritici*, while *mlo/ror2* double mutants display wild-type penetration rates (Peterhänsel et al., 1997).

In this study, we exploited available genomic information to perform a genome-wide characterization of tomato syntaxins. The functional role of two tomato syntaxins closely related to Arabidopsis PEN1, barley HvROR2, and grapevine VvPEN1 was investigated using RNA interference (RNAi) technology. Evolutionary analysis and multiple alignments were performed to identify molecular features putatively important for the function of the syntaxins.

## Materials and Methods

### Plant and Fungal Materials

The tomato *Slmlo1* line, carrying a loss-of-function mutation in the *SlMLO1* susceptibility gene (Bai et al., 2008; Zheng et al., 2016), and the tomato cultivar (cv) Moneymaker (MM) were used in the disease tests as resistant and susceptible control, respectively. The tomato *Slmlo1* line was also used as background genotype for RNAi experiments.

The Wageningen isolate of the tomato powdery mildew fungus *Oidium neolycopersici* and the Wag04 isolate of the barley powdery mildew fungus *Bgh*, maintained on the susceptible cvs. MM and Manchuria, respectively, were used for inoculation. Infected tomato and barley plants were kept in a greenhouse compartment at 20 ± 2°C with 70 ± 15% relative humidity at the Unifarm of Wageningen University & Research, Netherlands.

### Identification and Phylogenesis of Tomato Syntaxins

The Arabidopsis syntaxin AtPEN1 amino acid sequence (GenBank ID: NP_187788.1; gene AT3G11820) was used as query for a tBLASTn search against the Tomato Genome SL2.50 CDS of Sol Genomics Network (SGN), using default settings. The resulting 21 hits were used for a ClustalW alignment of 41 sequences, also including the complete Arabidopsis Qa SNARE family, grapevine VvPEN1, and barley HvROR2. The gap open cost and the gap extension cost were set equal to 10 and 4, respectively. Afterward, an Unweighted Pair Group Method with Arithmetic Mean (UPGMA) phylogenetic tree was built setting the bootstrap value equal to 100. All the bioinformatic analyses were performed using the CLC sequence viewer software^1^.

### Generation of Tomato RNAi Transformants

To generate RNAi constructs, the two tomato cDNA sequences showing the highest similarity to *AtPEN1*, Solyc10g081850.1.1 (named *SlPEN1a*) and Solyc01g006950.2.1 (named *SlPEN1b*), were used to design primer pairs for Gateway cloning (Invitrogen), using the Primer3 software (Untergasser et al., 2012). These primers, named *SlPEN1a*_gate__Fw/Rev (5′-caccCTGGTTGTGGACCTGGAAGT-3′/5′-TGTCCTCTTCCTTGCTCCTG-3′) and *SlPEN1b*_gate_Fw/Rev (5′-caccACGAGCTGAAAAACCTCGAA-3′/5′-ACA ACAGACGTCCTCGTCCT-3′), allowed the amplification of 259 and 250 bp PCR products for *SlPEN1a* and *SlPEN1b*, respectively. Amplification was conducted using *Pfu*UltraII Fusion HS DNA Polymerase (Agilent Technologies). PCR fragments were first cloned into the Gateway-compatible vector pENTR D-TOPO (Invitrogen) and then transferred to *Escherichia coli* competent cells (XL10-Gold Ultracompetent Cells, Agilent Technologies). The presence of the inserts was assessed by colony PCR, restriction enzyme digestion and sequencing using the universal M13 primers. Positive clones were used in a LR reaction, which allowed the inserts to be transferred to the pHELLSGATE12 vector (Helliwell and Waterhouse, 2003), harboring the 35S Cauliflower Mosaic Virus (CaMV) promoter for constitutive expression and the marker gene *NPTII* for kanamycin resistance selection. Plasmids were cloned into *E. coli* competent cells DH5α and positive colonies were screened by colony PCR and sequencing as described before. Recombinant vectors were finally extracted and transferred to the AGL1+virG strain of *Agrobacterium tumefaciens* by electroporation.

The tomato *Slmlo1* line was used for transformation, that was carried out as described by Appiano et al. (2015). A total of 15 T_1_ plants were generated and, after self-pollination, T_2_ families were obtained.

To select transgenic plants within each segregating family, the DNA of the T_2_ plants was amplified with primer pairs NPTII_Fw/ NPTII_Rev (5′-TCGGCTATGACTGGGCACAAC-3′)/5′-AAGAAGGCGATAGAAGGCGA-3′), and 35S-Fw/Rev (5′-GCTCCTACAAATGCCATCA-3′)/(5′-GATAGTGG GATTG TGCGTCA-3′). The expression of the target genes in selected T_2_ families was assessed by real-time qPCR using the primer pairs qPEN1a_Fw/Rev (5′-CGAGATGCTTTGTGCATCAG-3′/5′-CAGTCTCCTTCAGCTCCATTTC-3′) and qPEN1b_Fw/Rev (5′-TGGTTTAGTTGTTGATGGACCTC-3′/5′-ACCCCCATCCAACTTACTTACTTCTC-3′).

Transgenic T_2_ plants showing high silencing level for each construct were crossed and eight F_1_ individuals were obtained. Four-week-old F_1_ plants were tested through qPCR for the expression of *SlPEN1a* and *SlPEN1b*, using the primer pairs mentioned above.

### Disease Tests with *O. neolycopersici* and Quantification of Fungal Biomass

Three disease tests were performed using the adapted powdery mildew pathogen *O. neolycopersici*. The first one encompassed 20 plants for each of the 15 T_2_ families, seven *Slmlo1* plants and five MM plants. The second one included four T_2_ families (two carrying the RNAi::*SlPEN1a* construct and two carrying the RNAi::*SlPEN1b* construct), each including 15 transgenic plants, five non-transgenic plants and five *Slmlo1* plants. The third test included eight F_1_ individuals, 14 T_3_ transgenic individuals silenced for *SlPEN1a*, 19 T_3_ individuals silenced for *SlPEN1b*, and 8 *Slmlo1* individuals.

In all cases, the inoculation was performed by spraying plants with a suspension of conidiospores obtained from heavily infected leaves of MM plants and adjusted to a final concentration of 2^∗^10^4^ spores per milliliter, as described by Pavan et al. (2008). Inoculated plants were grown at 20 ± 2°C with 70 ± 15% relative humidity and day length of 16 h in a greenhouse of Unifarm of Wageningen University & Research, Netherlands.

Disease evaluation was carried out 15 days after inoculation. Powdery mildew symptoms were visually scored on each plant using a scale of symptoms severity ranging from 0 to 3 (Bai et al., 2008), and means and standard deviation were calculated for each T_2_ family. In addition, in the case of the second and third disease tests, fungal biomass was quantified by real-time qPCR as reported by Huibers et al. (2013). Briefly, plant and fungal genomic DNAs were isolated from infected tomato leaves using the Qiagen DNeasy Plant Mini Kit and amplified with the primer pairs On-Fw (5′-CGCCAAAGACCTAACCAAAA-3′)/On-Rev (5′-AGCCAAGAGATCCGTTGTTG-3′), designed on *O. neolycopersici* internal transcribed spacer (ITS) sequences (GenBank accession number EU047564), and Ef-Fw (5′-GGAACTTGAGAAGGAGCCTAAG-3′)/Ef-Rev (5′-CAACACCAACAGCAACAGTCT-3′), designed on the tomato *Elongation Factor* 1α (*Ef1*α) reference gene (Løvdal and Lillo, 2009). Relative fold-change of the ratio between fungus and tomato gDNAs was inferred by the 2^-ΔΔ*C*_T_^ method (Livak and Schmittgen, 2001; Pfaffl, 2001) and results were analyzed by the Student’s *t*-test.

### Disease Test with *Blumeria graminis* f. sp. *hordei* (*Bgh*) and Histological Analysis

Three transgenic plants from the T_2_ families RNAi::*SlPEN1a_*I and RNAi::*SlPEN1b_*I were selected by PCR using the 35S and NPTII primer pairs. Around 18 days after sowing, these plants, together with three plants of the *Slmlo1* line, were transferred to an infection chamber.

A dry inoculum of *Bgh* was brushed off heavily infected barley leaves with a paintbrush and applied on the third and fourth leaves of 4-week old tomato plants. At least three samples for each inoculated plant were collected 72 hours post-inoculation (hpi). These samples were stained with trypan blue as described by Freialdenhoven et al. (1996) and mounted on glass slides with a 1:1 (v/v) glycerol:water solution. Observation of the slides was done using a Zeiss Axiophot bright field microscope and pictures were taken with an Axiocam ERc5s. For each sample, more than thirty infection units (one infection unit representing a germinated spore) per slide were observed. The pathogen penetration rate was estimated as the percentage of units displaying hypersensitive response. Statistical analysis was carried out using the Student’s *t*-test.

### Detection of Conserved Syntaxin Motifs and Codon Evolutionary Analysis

To detect conserved motifs in the syntaxin family, the alignment used for phylogenetic analysis was given as input to the BOXSHADE software^2^, setting as 1.0 the fraction of sequences that must agree. Furthermore, with the aim of identifying residues specifically conserved in syntaxins acting in powdery mildew defense, another ClustalW alignment was performed, using proteins of the SYP-1b subclade. This new alignment was also fed to the BOXSHADE software.

Moreover, the same dataset was used for a codon-based evolutionary analysis, based on the difference of nonsynonymous-to-synonymous substitutions per non-synonymous and synonymous sites (dN-dS). The single-likelihood ancestor counting (SLAC) method implemented by the Datamonkey web server ^3^ was performed. The default *p*-value of 0.1 was used as threshold for statistical significance to make predictions on the kind of selection pressure (negative, neutral, or positive) acting on each codon.

## Results

### *In Silico* Identification of Tomato Syntaxins

**FIGURE 1:**
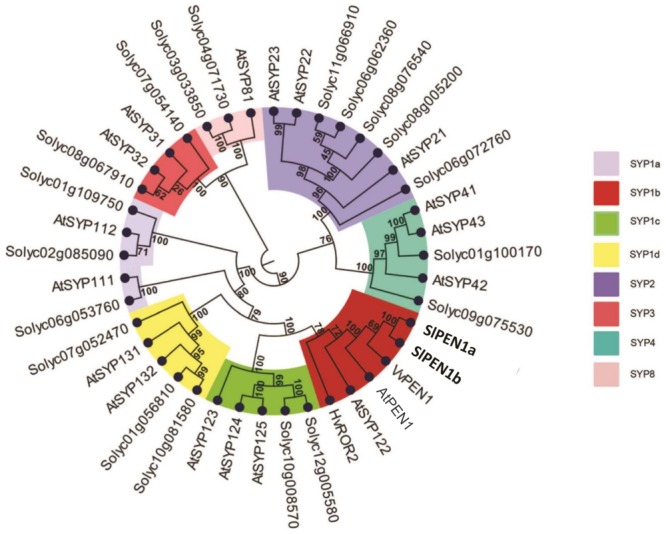
Phylogenetic relationships of 41 syntaxins. The dataset includes barley HvROR2, grapevine VvPEN1, the 18 Arabidopsis syntaxins (AtSYPs and AtPEN1) and the 21 predicted tomato syntaxins identified in this study (named with the SolGenomics Network database ID code). Numbers at nodes indicate bootstrap support values. Clades are named according to the nomenclature used for syntaxins subfamilies (SYP1, -2, -3, -4, -8). The clade SYP1 is further divided in four subclades (SYP1-a, -b, -c, and -d). The subclade SYP1b containing all the homologs known to be involved in plant–pathogen interactions (AtPEN1, AtSYP122, HvROR2, and VvPEN1) is highlighted in red.

Twenty-one putative syntaxins were retrieved in the tomato genome with a BLAST search using the Arabidopsis AtPEN1 amino acid sequence as input. These were used for a phylogenetic study, together with the 18 Arabidopsis syntaxins, barley HvROR2 and grapevine VvPEN1. The resulting phylogenetic tree was composed by five clearly distinct clades (**Figure 1**). The distribution of Arabidopsis syntaxins within the clades fully matched with their previous assignment to the five subfamilies SYP-1, -2, -3, -4, -8 (Sanderfoot et al., 2000). Within each clade, at least one tomato putative syntaxin was found. The most represented clade (SYP-1), harboring 21 homologs, was further partitioned into four subclades (indicated as SYP-1a to -1d in **Figure 1**).

Two putative tomato syntaxins, Solyc10g081850 and Solyc01g006950, were assigned with strong bootstrap support to the subclade SYP-1b, containing all the functional homologs shown to be involved in powdery mildew penetration resistance, i.e., AtPEN1, HvROR2, and VvPEN1 (Collins et al., 2003; Feechan et al., 2013). Therefore, Solyc10g081850 and Solyc01g006950 were renamed *SlPEN1a* and *SlPEN1b*, respectively (**Figure 1**). Protein sequences of SlPEN1a and SlPEN1b are highly related with each other (85.3% amino-acid conservation) and with AtPEN1 (72.3 and 71.7% amino-acid conservation, respectively).

### *SlPEN1a* Is Required for *mlo* Resistance to Tomato Powdery Mildew

To gain insights into the functional role of *SlPEN1a* and *SlPEN1b*, we obtained RNAi silencing constructs for each of the two genes and transformed the tomato *Slmlo1* line. Eleven RNAi::*SlPEN1a* and four RNAi::*SlPEN1b* T_1_ plants were obtained and self-pollinated to provide T_2_ families.

Two weeks after the inoculation, all the MM plants were covered by the fungal mycelium and had an average disease index (DI) score of 3 (Supplementary Table S1). Individuals of the *Slmlo1* line showed no symptoms on the 4th leaf. Since weak mycelium growth was occasionally observed on the first three true leaves, an average DI score of 0.62 was assigned to them.

The 11 T_2_ families obtained with the RNAi::*SlPEN1a* construct showed various levels of symptoms corresponding to average DI scores ranging from 0.47 to 1.49. The four T_2_ families obtained with the RNAi::*SlPEN1b* construct displayed average DI scores ranging from 0.8 to 0.95.

**FIGURE 2:**
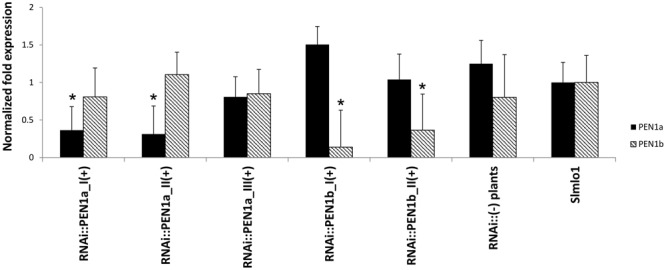
Relative expression of *SlPEN1a* and *SlPEN1b* in RNAi transformants obtained from the genetic background of the *Slmlo1* line. Data refer to 15 transgenic individuals of three T_2_ families (I, II, and III) for the RNAi::*SlPEN1a* silencing construct and two T_2_ families (I and II) for the RNAi::*SlPEN1b*. The columns indicated as RNAi::(-) plants and *Slmlo1* refer to five individuals, respectively. Bars indicate the standard errors. Asterisks indicate significant differences with the *Slmlo1* line, inferred by Student’s *t*-test (*p* < 0.05).

Individuals of five selected T_2_ families associated with the highest average DI scores (namely RNAi::PEN1a_I, RNAi::PEN1a_II, RNAi::PEN1a_III, RNAi::*PEN1b*_I, and RNAi::*PEN1b*_II), were screened for the presence/absence of the construct. Plants carrying the RNAi constructs were identified and further referred to as RNAi::*SlPEN1a*(+) and RNAi::*SlPEN1b*(+). A higher average DI score was assigned to them compared to T_2_ plants not harboring the corresponding RNAi constructs (Supplementary Table S1). To verify that higher susceptibility of individuals carrying the RNAi constructs was due to reduced *SlPEN1a* and *SlPEN1b* transcripts, the expression level of these genes was evaluated. The *SlPEN1a* homolog was significantly silenced in transgenic individuals of the families RNAi::*SlPEN1a*_I(+) and RNAi::*SlPEN1a*_II(+) compared to *Slmlo1* plants. In addition, no unwanted cross-silencing of *SlPEN1b* was found (**Figure 2**). Transgenic plants identified in family RNAi::*SlPEN1a*_III did not show a significant silencing of the target gene. However, this family was the least susceptible of the three T_2_ families carrying the RNAi::*SlPEN1a* construct. The *SlPEN1b* homolog was strongly silenced in transgenic plants of families RNAi::*PEN1b*_I and _II compared to *Slmlo1* plants, while the expression of *SlPEN1a* was not significantly reduced.

A total of five non-transgenic individuals segregating from the five T_2_ families were grouped to evaluate the expression of *SlPEN1a* and *SlPEN1b*. The expression of these genes did not significantly change compared to *Slmlo1* plants (**Figure 2**).

**FIGURE 3:**
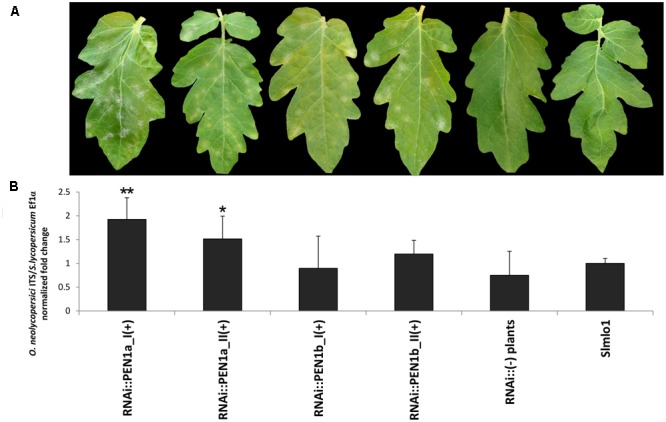
Effect of RNAi silencing of tomato *SlPEN1a* and *SlPEN1b* in the genetic background of the *Slmlo1* line on the growth of *Oidium neolycopersici*. **(A)** Shows the phenotype of leaves collected 15 days after pathogen inoculation. From left to right: selected transgenic individuals of two independent T_2_ families carrying the *SlPEN1a* silencing construct [RNAi::*SlPEN1a*_I(+) and RNAi::*SlPEN1a*_II(+)], selected individuals of two independent T_2_ families carrying the *SlPEN1b* silencing construct [RNAi::*SlPEN1b*_I(+) and RNAi::*SlPEN1b*_II(+)], a non-transgenic plant segregating from one of the T_2_ families, and an individual of the *Slmlo1* resistant line. **(B)** Refers to the relative quantification of the ratio between *O. neolycopersici* and tomato gDNAs in 15 transgenic individuals of the same families above mentioned, five non-transgenic T_2_ individuals, and five *Slmlo1* individuals. Bars indicate the standard errors. Asterisks refer to significant differences with the *Slmlo1* plants, inferred by Student’s *t*-test (^∗^*p* < 0.05; ^∗∗^*p* < 0.01).

Aimed to confirm our results, the four families showing high silencing level of the target genes (RNAi::*PEN1a*_I and _II; RNAi::*PEN1b*_I and _II) were selected for a second disease test (**Figure 3**). Compared to the *Slmlo1* plants, RNAi::*SlPEN1a*(+) individuals showed once more clear fungal sporulation (**Figure 3A**) and significantly increased fungal biomass (**Figure 3B**). Although RNAi::*SlPEN1b*(+) individuals showed slightly higher DI than *Slmlo1* plants, fungal biomass did not significantly increase (**Figure 3B**). Non-transgenic individuals of the four T_2_ families displayed the same DI of *Slmlo1* individuals (**Figure 3**).

**FIGURE 4:**
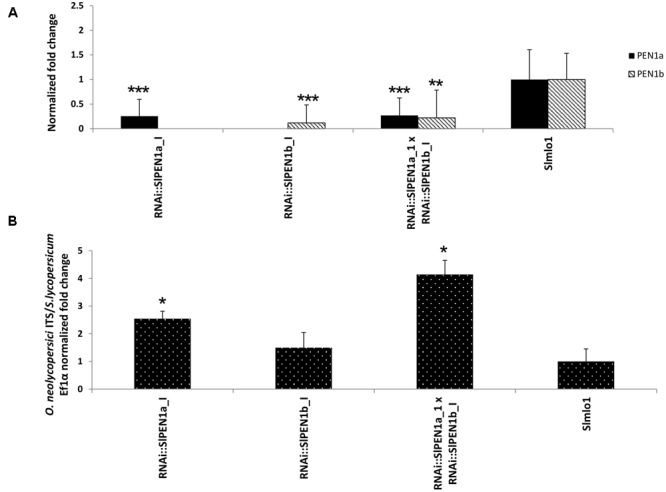
Effect of silencing both *SlPEN1a* and *SlPEN1b* [RNAi::*SlPEN1a_*I(+) × RNAi::*SlPEN1b*_I(+)] in the genetic background of the *Slmlo1* line on the growth of *O. neolycopersici*. **(A)** Shows the expression of *SlPEN1a* and *SlPEN1b* evaluated on 14 individuals carrying the RNAi::*SlPEN1a*_I construct, 19 carrying the RNAi::*SlPEN1b*_I and eight individuals of the F_1_ progeny RNAi::*SlPEN1a*_I (+) × RNAi::*SlPEN1b*_I(+), compared to eight *Slmlo1* plants. **(B)** Shows the quantification of fungal growth on the same dataset of plants. In both panels, bars indicate standard errors. Asterisks refer to significant differences, inferred by Student’s *t*-test (^∗^*p* < 0.05; ^∗∗^*p* < 0.01; ^∗∗∗^*p* < 0.001).

To further investigate the functional role of *SlPEN1b* and its relation with *SlPEN1a*, we performed an additional experiment using the F_1_ progeny obtained by crossing well-silenced RNAi::*SlPEN1a*_I(+) and RNAi::*SlPEN1b*_I(+) individuals. Significantly reduced expression level of the target gene was observed in the eight F_1_ individuals and the selfing progenies of their parents, which were included in the same experiment (**Figure 4**). Compared to the control group of eight *Slmlo1* plants, the F_1_ progeny and RNAi::*SlPEN1a*(+) plants showed a significantly higher fungal biomass. However, F_1_ individuals were not significantly more susceptible than plants carrying the RNAi::*SlPEN1a*(+) construct (data not shown).

### Functional Characterization of *SlPEN1a* and *SlPEN1b* in a Non-host Interaction

**FIGURE 5:**
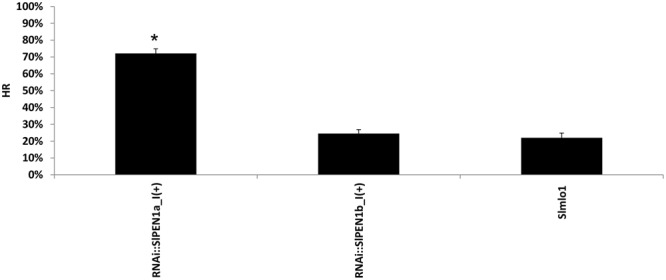
Effect of RNAi silencing of tomato *SlPEN1a* and *SlPEN1b* on the interaction with the non-adapted powdery mildew fungus *Blumeria graminis* f. sp. *hordei (Bgh)*. The graph shows the percentage of infection units causing hypersensitive response on the *Slmlo1* line and on transgenic individuals silenced for *SlPEN1a* and *SlPEN1b* [RNAi::*SlPEN1a_*I(+) and RNAi::*SlPEN1b*_I(+)]. Bars refer to standard errors calculated on three biological replicates. Asterisks indicate significant difference with the *Slmlo1* line inferred by the Student’s *t*-test (*p* ≤ 0.05).

Since it has been shown that *PEN* genes are involved in resistance to non-adapted powdery mildew species, we set-up an assay to investigate the role of the two tomato syntaxins, *SlPEN1a* and *SlPEN1b*, in the interaction with barley powdery mildew fungus *Bgh.* An artificial inoculum of this pathogen was used to inoculate RNAi::*SlPEN1a*_I(+) and RNAi::*SlPEN1b*_I(+) individuals, together with *Slmlo1* plants. An histological analysis was performed to observe if pathogen entry rate changed in these transgenic plants. We noticed that the percentage of infection units showing HR increased dramatically from 22 to 72% in RNAi::*SlPEN1a*-I(+) individuals compared to the *Slmlo1* line (**Figure 5**). A slight increase (2.5%) of HR was also found in RNAi::*SlPEN1b*_I(+) plants, although this value was not statistically different from the one of the *Slmlo1* line (**Figure 5**). The increased HR rate in RNAi::*SlPEN1a*_I(+) plants suggests that silencing *SlPEN1a* favors *Bgh* penetration. Indeed, HR has been assumed to act as a post-penetration defense mechanism in non-host interactions (Lipka et al., 2005).

### Identification of Molecular Features Putatively Required for Syntaxin Function

**FIGURE 6:**
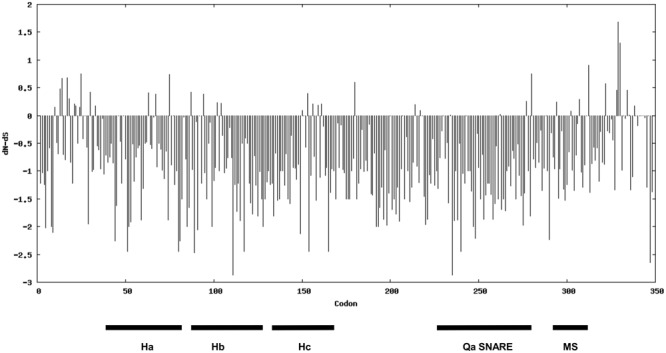
Direction and magnitude of natural selection acting on the syntaxins belonging to the SYP1b subclade. The bars indicate dN-dS values. The position of characteristic domains of syntaxins (the three helix domain Ha, Hb and Hc, the Qa-SNARE domain and the membrane-spanning (MS) domain are indicated.

Aiming to investigate the magnitude and direction of natural selection acting on syntaxins involved in defense against powdery mildew fungi, we used the nucleotide sequence of SYP-1b homologs for a codon-based SLAC evolutionary analysis. This analysis is based on the dissimilarity level between non-synonymous substitution (dN) and synonymous substitution (dS) values. Evidence for negative selection was found on 77 codons, associated with amino acid residues scattered in various syntaxin domains (Qa-SNARE, membrane-spanning, and the three auto-inhibitory helix domains known as Ha, Hb, Hc) (**Figure 6** and Supplementary Table S2). Alignment of the SYP-1b subclade proteins revealed that 65 of these residues are invariable throughout the dataset, suggesting they might play a crucial role for protein function.

We performed an additional alignment with the same set of syntaxins used for phylogenetic analysis. In total, we found that 24 out of 65 residues that are invariable in the SYP-1b subclade are not conserved in other syntaxins, thus pointing to the identification of residues that are, possibly, specifically important for pathogen defense (Supplementary Figure S1). In addition, we noticed two amino acid differences (highlighted in red) between the proteins playing a major role in penetration defense (AtPEN1/SlPEN1a/VvPEN1/HvROR2) and the other two proteins of subclade SYP-1b (AtSYP122/SlPEN1b) (Supplementary Figure S2).

## Discussion

### The Tomato Syntaxin Family

In this study, a genome-wide survey allowed the identification of 21 syntaxins in the tomato genome. The number of tomato homologs is consistent with similar genome-wide studies showing that *A. thaliana* contains 18 syntaxins, *O. sativa* 14 and *P. trichocarpa* 22 (International Rice Genome Sequencing Project, 2005; Tuskan et al., 2006; Lipka et al., 2007). The identification of 10 putative tomato SYP1 members corroborates previous findings indicating that this subfamily, containing homologs localized in the plasma membrane, is more represented than the other subfamilies SYP2, -3, -4, and -8 in plants (Lipka et al., 2007) (Supplementary Table S3).

It is known that specific SYP1 homologs in Arabidopsis are involved in other physiological processes besides defense against pathogens. Specifically, SYP111 controls cytokinesis (Lauber et al., 1997), whereas SYP123 and SYP132 mediate root hair tip growth (Ichikawa et al., 2014). Furthermore, the SYP132 ortholog in *Medicago truncatula* homolog (MtSYP132) and *Nicotiana benthamiana* (NtSYP132) were found to be involved in the symbiosome formation of *Sinorhizobium meliloti* and *Pseudomonas syringae* resistance, respectively (Catalano et al., 2007; Kalde et al., 2007; Limpens et al., 2009). Phylogenetic analysis presented in this study suggests that the tomato syntaxin Solyc06g053760 is an ortholog of Arabidopsis SYP111. Evolutionary relatedness between SYP132 and a couple of tomato syntaxins, Solyc01g056810 and Solyc10g081580, might indicate that these genes are SYP132 co-orthologs due to a recent duplication event after the split of two lineages from an Arabidopsis-tomato common ancestor.

### Involvement of a Tomato Syntaxin in Plant-PM Pathogen Interaction

Two tomato syntaxins, SlPEN1a and SlPEN1b, are grouped in the phylogenetic clade also containing homologs previously reported to play a major (AtPEN1, HvROR2, and VvPEN1) or minor (AtSYP122) role in penetration resistance to powdery mildew fungi (**Figure 1**). Silencing of *SlPEN1a* compromised penetration resistance to both adapted and non-adapted powdery mildew pathogens in the *Slmlo1* tomato line, showing that *SlPEN1a* is likely the functional ortholog of *HvROR2, AtPEN1* and *VvPEN1.* This information might be useful for tomato breeding and for future studies on plant–pathogen interaction using tomato as model organism.

Similarly to barley, we found that the impairment of the tomato *SlPEN1a* syntaxin in the *Slmlo1* background macroscopically restored the susceptibility to the powdery mildew disease. In contrast, the *Atmlo2pen1* double mutant, although being characterized by wild-type levels of fungal penetration, still displayed a macroscopically resistant phenotype (Consonni et al., 2006). AtPEN1 was shown to act as a negative regulator of the hormone salicylic acid, known to enhance post-penetration defense mechanisms against biotrophic (Zhang et al., 2007). Possibly, differently from AtPEN1, SlPEN1a and barley HvROR2 have minor or no role in the regulation of salicylic-acid-mediated defense pathways. However, further testing needs to be done to support this hypothesis.

The *Atpen1Atsyp122* double knock-out mutant displays a severely stunted and necrotic phenotype (Assaad et al., 2004). In the present study, transgenic individuals, silencing both *SlPEN1a* and *SlPEN1b* homologs, were devoid of markedly visible pleiotropic effects which might be due to residual gene expression. Alternatively, they may not affect the growth and development of the plant as their corresponding Arabidopsis orthologs. Also in this case, further tests are required to prove this hypothesis.

### Amino Acids of Syntaxins Playing a Potential Role in Penetration Resistance

In this work, a codon-based evolutionary analysis allowed us to detect 77 codons under significant negative selection in the SYP-1b subclade, containing the syntaxins associated with defense against powdery mildew fungi. Moreover, alignment of all the 41 syntaxin sequences pointed out the occurrence of 24 residues specifically conserved in the SYP-1b subclade. Three of these residues are located in the Qa-SNARE domain, whereas 10 are found in the auto-inhibitory motifs Ha, Hb or Hc helices of Qa-SNARE proteins. These helices, when folded in a closed conformation, prevent the exposure of the Qa-SNARE domain and thus the formation of SNARE complexes (Collins et al., 2003). In barley *ror2* mutant, a deletion of 31 amino acids (S118-E148) of the Hc helix occurred, which might have led to a constitutively open state of the protein resulting in enhanced binding to HvSNAP34 (Collins et al., 2003). Interestingly, the deletion contains four of the 24 conserved residues identified in this study, namely G123, P124, T133 and G138, in HvROR2 sequence, suggesting an important function of these residues.

Besides the above discussed 24 amino acids specifically conserved in the SYP-1b subclade, we pointed out two different amino acids present in AtSYP122 and SlPEN1b but not in any of the four SYP1b syntaxins with well-characterized role in plant–pathogen interactions, namely A111 and I286 (Supplementary Figure S2). In the study of Pajonk et al. (2008), authors obtained a chimeric syntaxin by swapping the first 175 amino acids of the AtPEN1 N-terminal domain (including the residue A111), with the corresponding sequence of AtSYP122. The construct was then used to transform *pen1* mutant. The authors did not observe any difference in the level of *Bgh* entry rate between the obtained transformants and their corresponding genetic background. This observation suggests that N-terminal region of the AtPEN1 protein contains amino acids of critical importance for its functionality, possibly including the A111 residue mentioned above. Future functional analyses, aimed at targeting these amino acids, might unravel their importance toward the functional specialization observed between AtPEN1/ SlPEN1a/VvPEN1/HvROR2 and AtSYP122/SlPEN1b.

## Author Contributions

Conceived and designed the experiments: MA, VB, SP, and YB. Performed the experiments: VB, MA, ZY, and ZZ. Analyzed the data: MA, VB, and SP. Contributed reagents/materials/ analysis tools: LR and RV. Wrote and edited the paper: VB, MA, SP, A-MW, RV, and YB.

## Conflict of Interest Statement

The authors declare that the research was conducted in the absence of any commercial or financial relationships that could be construed as a potential conflict of interest. The reviewer PS-B and handling Editor declared their shared affiliation.

## Footnotes

1 http://www.clcbio.com/

2 http://www.ch.embnet.org/software/BOX_form.html

3 www.datamonkey.org

## References

[B1] Appiano M. Pavan S. Catalano D. Zheng Z. Bracuto V. Lotti C. (2015). Identification of candidate *MLO* powdery mildew susceptibility genes in cultivated Solanaceae and functional characterization of tobacco *NtMLO1*. *Transgenic Res.* 24 847–858. 10.1007/s11248-015-9878-4 25947088 PMC4569668

[B2] Assaad F. Qiu J. L. Youngs H. Ehrhardt D. Zimmerli L. Kalde M. (2004). The *PEN1* syntaxin defines a novel cellular compartment upon fungal attack and is required for the timely assembly of papillae. *Mol. Biol. Cell* 15 5118–5129. 10.1091/mbc.E04-02-0140 15342780 PMC524786

[B3] Bai Y. Pavan S. Zheng Z. Zappel N. F. Reinstädler A. Lotti C. (2008). Naturally occurring broad-spectrum powdery mildew resistance in a Central American tomato accession is caused by loss of *Mlo* function. *Mol. Plant Microbe Interact.* 21 30–39. 10.1094/MPMI-21-1-0030 18052880

[B4] Bock J. B. Matern H. T. Peden A. A. Scheller R. H. (2001). A genomic perspective on membrane compartment organization. *Nature* 409 839–841. 10.1038/35057024 11237004

[B5] Catalano C. M. Czymmek K. J. Gann J. G. Sherrier D. J. (2007). *Medicago truncatula* syntaxin SYP132 defines the symbiosome membrane and infection droplet membrane in root nodules. *Planta* 225 541–550. 10.1007/s00425-006-0369-y 16944200

[B6] Collins N. C. Thordal-Christensen H. Lipka V. Bau S. Kombrink E. Qiu J. L. (2003). SNARE-protein-mediated disease resistance at the plant cell wall. *Nature* 425 973–977. 10.1038/nature02076 14586469

[B7] Consonni C. Humphry M. E. Hartmann H. A. Livaja M. Durner J. Westphal L. (2006). Conserved requirement for a plant host cell protein in powdery mildew pathogenesis. *Nat. Genet.* 38 716–720. 10.1038/ng1806 16732289

[B8] Fasshauer D. Sutton R. B. Brunger A. T. Jahn R. (1998). Conserved structural features of the synaptic fusion complex: SNARE proteins reclassified as Q- and R-SNAREs. *Proc. Natl. Acad. Sci. U.S.A.* 95 15781–15786. 10.1073/pnas.95.26.15781 9861047 PMC28121

[B9] Feechan A. Jermakow A. M. Ivancevic A. Godfrey D. Pak H. Panstruga R. (2013). Host cell entry of powdery mildew is correlated with endosomal transport of antagonistically acting *VvPEN1* and *VvMLO* to the papilla. *Mol. Plant Microbe Interact.* 26 1138–1150. 10.1094/MPMI-04-13-0091-R 23819806

[B10] Freialdenhoven A. Peterhansel C. Kurth J. Kreuzaler F. Schulze-Lefert P. (1996). Identification of genes required for the function of non-race-specific *mlo* resistance to powdery mildew in barley. *Plant Cell* 8 5–14. 10.1105/tpc.8.1.5 12239354 PMC161077

[B11] Helliwell C. Waterhouse P. (2003). Constructs and methods for high-throughput gene silencing in plants. *Methods* 30 289–295. 10.1016/S1046-2023(03)00036-7 12828942

[B12] Huibers R. P. Loonen A. E. H. M. Gao D. Van den Ackerveken G. Visser R. G. F. Bai Y. (2013). Powdery mildew resistance in tomato by impairment of *SlPMR4* and *SlDMR1*. *PLOS ONE* 8:e67467 10.1371/journal.pone.0067467 PMC3688610 23818978

[B13] Ichikawa M. Hirano T. Enami K. Fuselier T. Kato N. Kwon C. (2014). Syntaxin of plant proteins *SYP123* and *SYP132* mediate root hair tip growth in *Arabidopsis thaliana*. *Plant Cell Physiol.* 55 790–800. 10.1093/pcp/pcu048 24642714

[B14] International Rice Genome Sequencing Project (2005). The map-based sequence of the rice genome. *Nature* 436 793–800. 10.1038/nature03895 16100779

[B15] Kalde M. Nühse T. S. Findlay K. Peck S. C. (2007). The syntaxin SYP132 contributes to plant resistance against bacteria and secretion of pathogenesis-related protein 1. *Proc. Natl. Acad. Sci. U.S.A.* 104 11850–11855. 10.1073/pnas.0701083104 17592123 PMC1913864

[B16] Lauber M. H. Waizenegger I. Steinmann T. Schwarz H. Mayer U. Hwang I. (1997). The Arabidopsis KNOLLE protein is a cytokinesis-specific syntaxin. *J. Cell Biol.* 139 1485–1493. 10.1083/jcb.139.6.1485 9396754 PMC2132613

[B17] Limpens E. Ivanov S. van Esse W. Voets G. Fedorova E. Bisseling T. (2009). Medicago N(2)-fixing symbiosomes acquire the endocytic identity marker Rab7 but delay the acquisition of vacuolar identity. *Plant Cell* 21 2811–2828. 10.1105/tpc.108.064410 19734435 PMC2768938

[B18] Lipka V. Dittgen J. Bednarek P. Bhat R. Wiermer M. Stein M. (2005). Plant science: Pre- and postinvasion defenses both contribute to nonhost resistance in Arabidopsis. *Science* 310 1180–1183. 10.1126/science.1119409 16293760

[B19] Lipka V. Kwon C. Panstruga R. (2007). SNARE-ware: the role of SNARE-domain proteins in plant biology. *Annu. Rev. Cell Dev. Biol.* 23 147–174. 10.1146/annurev.cellbio.23.090506.123529 17506694

[B20] Livak K. J. Schmittgen T. D. (2001). Analysis of relative gene expression data using real-time quantitative PCR and the 2^-ΔΔ*C*_T_^ Method. *Methods* 25 402–408. 10.1006/meth.2001.1262 11846609

[B21] Løvdal T. Lillo C. (2009). Reference gene selection for quantitative real-time PCR normalization in tomato subjected to nitrogen, cold, and light stress. *Anal. Biochem.* 387 238–242. 10.1016/j.ab.2009.01.024 19454243

[B22] Pajonk S. Kwon C. Clemens N. Panstruga R. Schulze-Lefert P. (2008). Activity determinants and functional specialization of Arabidopsis PEN1 syntaxin in innate immunity. *J. Biol. Chem.* 283 26974–26984. 10.1074/jbc.M805236200 18678865

[B23] Pavan S. Jacobsen E. Visser R. G. Bai Y. (2010). Loss of susceptibility as a novel breeding strategy for durable and broad-spectrum resistance. *Mol. Breed.* 25 1–12. 10.1007/s11032-009-9323-6 20234841 PMC2837247

[B24] Pavan S. Zheng Z. Borisova M. Van Den Berg P. Lotti C. De Giovanni C. (2008). Map- vs. homology-based cloning for the recessive gene *ol-2* conferring resistance to tomato powdery mildew. *Euphytica* 162 91–98. 10.1007/s10681-007-9570-8

[B25] Peterhänsel C. Freialdenhoven A. Kurth J. Kolsch R. Schulze-Lefert P. (1997). Interaction analyses of genes required for resistance responses to powdery mildew in barley reveal distinct pathways leading to leaf cell death. *Plant Cell* 9 1397–1409. 10.1105/tpc.9.8.1397 12237388 PMC157006

[B26] Pfaffl M. W. (2001). A new mathematical model for relative quantification in real-time RT-PCR. *Nucleic Acids Res.* 29 e45 10.1093/nar/29.9.e45 PMC55695 11328886

[B27] Sanderfoot A. A. Assaad F. F. Raikhel N. V. (2000). The Arabidopsis genome. An abundance of soluble N-ethylmaleimide-sensitive factor adaptor protein receptors. *Plant Physiol.* 124 1558–1569. 10.1104/pp.124.4.1558 11115874 PMC59855

[B28] Schilde C. Lutter K. Kissmehl R. Plattner H. (2008). Molecular identification of a SNAP-25-like SNARE protein in Paramecium. *Eukaryot. Cell* 7 1387–1402. 10.1128/EC.00012-08 18552286 PMC2519768

[B29] Seifi A. Gao D. Zheng Z. Pavan S. Faino L. Visser R. F. (2014). Genetics and molecular mechanisms of resistance to powdery mildews in tomato (*Solanum lycopersicum*) and its wild relatives. *Eur. J. Plant Pathol.* 138 641–665. 10.1007/s10658-013-0314-4

[B30] Söllner T. Whiteheart S. W. Brunner M. Erdjument-Bromage H. Geromanos S. Tempst P. (1993). SNAP receptors implicated in vesicle targeting and fusion. *Nature* 362 318–324. 10.1038/362318a0 8455717

[B31] Takamatsu S. (2004). Phylogeny and evolution of the powdery mildew fungi (Erysiphales, Ascomycota) inferred from nuclear ribosomal DNA sequences. *Mycoscience* 45 147–157. 10.1007/S10267-003-0159-3

[B32] Trujillo M. Troeger M. Niks R. E. Kogel K. H. Hückelhoven R. (2004). Mechanistic and genetic overlap of barley host and non-host resistance to *Blumeria graminis*. *Mol. Plant Pathol.* 5 389–396. 10.1111/j.1364-3703.2004.00238.x 20565615

[B33] Tuskan G. A. Difazio S. Jansson S. Bohlmann J. Grigoriev I. Hellsten U. (2006). The genome of black cottonwood, *Populus trichocarpa* (Torr. & Gray). *Science* 313 1596–1604. 10.1126/science.1128691 16973872

[B34] Untergasser A. Cutcutache I. Koressaar T. Ye J. Faircloth B. C. Remm M. (2012). Primer3—new capabilities and interfaces. *Nucleic Acids Res.* 40 e115 10.1093/nar/gks596 PMC3424584 22730293

[B35] Zhang Z. Feechan A. Pedersen C. Newman M. A. Qiu J. Olesen K. L. (2007). A SNARE-protein has opposing functions in penetration resistance and defence signalling pathways. *Plant J.* 49 302–312. 10.1111/j.1365-313X.2006.02961.x 17241452

[B36] Zheng Z. Appiano M. Pavan S. Bracuto V. Ricciardi L. Visser R. G. F. (2016). Genome-wide study of the tomato *SlMLO* gene family and its functional characterization in response to the powdery mildew fungus *Oidium neolycopersici*. *Front. Plant Sci.* 7:380 10.3389/fpls.2016.00380 PMC4986958 27579028

