# Supplementary material for: Functional Characterization of a Syntaxin Involved in Tomato (Solanum lycopersicum) Resistance against Powdery Mildew
Source: Front Plant Sci. 2017 Sep 20;8:1573. doi: 10.3389/fpls.2017.01573 (PMC5611543; doi:10.3389/fpls.2017.01573)
Supplement: Supplementary file 1 [file Data_Sheet_1.PDF]

Extra

Functional characterization of a tomato syntaxin involved in powdery mildew resistance

Table S1 Average disease index (DI) scores visually assigned to 11 and four T_2 families obtained after transforming the *Slmlo1* line with the RNAi::*SlPEN1a* and RNAi::*SlPEN1b* constructs. In addition, the average DI scores assigned to the susceptible tomato Moneymaker and the resistant *Slmlo1* line are listed. NPTII(+) and NPTII(-) indicate T_2 plants carrying the corresponding RNAi construct (+) or not (-).

**Family label**	**Average DI score ± st dev**	**Average DI score ± st dev NPTII(+)**	**Average DI score ± st dev NPTII (-)**
RNAi::SlPEN1a_I	1.49 ± 0.52	1.73 ± 0.32	0.75 ± 0.17
RNAi::SlPEN1a_II	1.11 ± 0.39	1.22 ± 0.32	0.5 ± 0
RNAi::SlPEN1a_III	0.91 ± 0.29	1.06 ± 0.28	0.75 ± 0
RNAi::SlPEN1a_IV	0.84 ± 0.34		
RNAi::SlPEN1a_V	0.47 ± 0.11		
RNAi::SlPEN1a_VI	0.52 ± 0.16		
RNAi::SlPEN1a_VII	0.89 ± 0.26		
RNAi::SlPEN1a_VIII	0.69 ± 0.16		
RNAi::SlPEN1a_IX	0.76 ± 0.26		
RNAi::SlPEN1a_X	0.59 ± 0.12		
RNAi::SlPEN1a_XI	0.54 ± 0.09		
RNAi::SlPEN1b_I	0.95 ± 0.27	1.06 ± 0.28	0.78 ± 0.16
RNAi::SlPEN1b_II	0.82 ± 0.31	0.95 ± 0.35	0.65 ± 0.22
RNAi::SlPEN1b_III	0.8 ± 0.3		
RNAi::SlPEN1b_IV	0.82 ± 0.14		
Moneymaker	3		
Slmlo1	0.62 ± 0.35		

Table S2 Residues under significant negative selection in SYP1b syntaxins. Residue numbers refer to amino acid position in the AtPEN1 protein. DN-dS values, corresponding p-values and residue position with respect to characteristic syntaxin domains (the three helix domain Ha-Hb- Hc, the Qa-SNARE domain and the membrane-spanning (MS) domain are reported.

Corresponding residue in AtPEN1	dN-dS	p-value	Domain
**F5**	-3.15883	0.06131	-
**S8**	-3.1351	0.02213	-
**F9**	-3.28782	0.09351	-
**N43**	-3.52804	0.05074	Ha
**L44**	-2.54257	0.05726	Ha
**D50**	-3.83057	0.01861	Ha
**V51**	-3.1351	0.01235	Ha
**E52**	-3.00033	0.03562	Ha
**L58**	-2.94697	0.01581	Ha
**E73**	-2.94552	0.03696	Ha
**H79**	-3.83057	0.01861	Ha
**N80**	-3.52804	0.05074	-
**A81**	-2.35132	0.03704	-
**V84**	-3.1351	0.01235	-
**R88**	-3.87355	0.01415	Hb
**K90**	-3.21478	0.06364	Hb
**V95**	-2.35132	0.03704	Hb
**A98**	-3.1351	0.01235	Hb
**E110**	-4.49517	0.00675	Hb
**L112**	-2.69996	0.02732	Hb

**R114**	-2.95551	0.01719	Hb
**N116**	-3.83057	0.01861	Hb
**R120**	-2.46381	0.03418	Hb
**G124**	-2.8307	0.04851	Hb
**G126**	-2.35132	0.0434	-
**P127**	-3.1351	0.01235	-
**G128**	-2.35132	0.03841	-
**R133**	-2.8257	0.02516	Hc
**R135**	-2.39318	0.07344	Hc
**T136**	-2.35132	0.03704	Hc
**G141**	-2.35132	0.03868	Hc
**L142**	-2.48174	0.03486	Hc
**L146**	-1.80166	0.08323	Hc
**D148**	-3.33705	0.05724	Hc
**F153**	-3.83057	0.01861	Hc
**R157**	-2.39325	0.07349	Hc
**Y164**	-3.83057	0.02885	Hc
**V168**	-2.35132	0.03704	-
**T174**	-2.35132	0.03704	-
**V175**	-2.35132	0.03704	-
**T176**	-2.35132	0.03704	-
**P180**	-2.35132	0.03704	-

**L188**	-2.21353	0.04439	-
**T191**	-3.1351	0.01235	-
**G192**	-3.1351	0.01249	-
**E193**	-2.58975	0.09884	-
**E195**	-2.93078	0.03733	-
**F197**	-3.08635	0.06377	-
**L198**	-2.18715	0.05751	-
**A201**	-2.35132	0.03704	-
**I202**	-2.77566	0.03544	-
**E204**	-2.9842	0.03601	-
**G206**	-2.35132	0.04295	-
**I217**	-2.27694	0.05266	-
**E219**	-2.92783	0.03741	-
**R220**	-1.6729	0.09756	-
**L231**	-2.45666	0.06387	Qa SNARE
**E233**	-4.49517	0.00675	Qa SNARE

**L234**	-2.95785	0.01765	Qa SNARE
**Q236**	-2.94552	0.04219	Qa SNARE
**F238**	-3.83057	0.01861	Qa SNARE
**L244**	-2.13677	0.06406	Qa SNARE
**V245**	-3.1351	0.01235	Qa SNARE
**E246**	-3.45719	0.06013	Qa SNARE
**G249**	-2.35132	0.04494	Qa SNARE
**Q251**	-2.92688	0.04273	Qa SNARE
**L252**	-2.24543	0.04253	Qa SNARE
**I255**	-2.22416	0.05519	Qa SNARE
**E256**	-2.93077	0.03733	Qa SNARE
**V259**	-2.35132	0.03704	Qa SNARE
**R261**	-2.64477	0.03867	Qa SNARE
**A262**	-2.35132	0.03704	Qa SNARE
**G269**	-2.35132	0.04325	Qa SNARE
**L273**	-3.09493	0.0138	Qa SNARE

**R277**	-2.82596	0.0199	Qa SNARE
**R284**	-2.12025	0.06354	-
**C288**	-3.49295	0.06064	Membrane-spanning

Table S3 Distribution of *Arabidopsis thaliana*, *Oryza sativa*, *Populus trichocarpa* and *Solanum lycopersicum* syntaxins in subfamilies.

SNARE type	SNARE subfamily	*A. thaliana*	*O. sativa*	*P. trichocarpa*	*S.lycopersicum*
Qa SNARE	SYP1	9	7	11	10
	SYP2	3	3	3	5
	SYP3	2	1	3	2
	SYP4	3	1	3	2
	SYP8	1	2	2	2
	tot	**18**	**14**	**22**	**21**

```
AtPEN1           1 MN--------------------DLFSSSFSRFRSGEPSPRRDVAGGGD-GVQMANPAGST
VvPEN1           1 MN--------------------DLFSGSFSRFRSEEPPPS----------VEMTS---ST
HvROR2           1 MN--------------------NLFSSSWKRA---GAGGDGDLESGGG-GVEMTAPPGAA
SlPEN1a          1 MN--------------------DLFSGSFSRFRNEEQSPNQESAG-----IQMRQ---QT
SlPEN1b          1 MN--------------------DLFSGSFSRYR--ENDHDQDSHG-----IEMGD----T
AtSYP122         1 MN--------------------DLLSGSFKTSVADGSSPPHS-----H-NIEMSKAKVSG
AtSYP124         1 MN--------------------DLFSSSFKKYTDLKQQAQMD-------DIESGK-----
AtSYP123         1 MN--------------------DLISSSFKRYTDLNHQVQLD-------DIESQNVSL--
AtSYP111         1 MN--------------------DLMTKSFMSYVDLKKAAMKDMEAGPDFDLEMAS----T
AtSYP131         1 MN--------------------DLLKGSLEFSRDR---SNRS-------DIESG-HGPGN
AtSYP125         1 MN--------------------DLFSNSFKK-----NQAQLG-------DVEAGQ-----
AtSYP132         1 MN--------------------DLLKGS--FELPRGQSSREG-------DVELGEQ---Q
AtSYP112         1 MN--------------------DLMTKSFLSYVELKKQARTDMES--DRDLEKGEDFNFD
Solyc12g005580   1 MN--------------------DLFSNSFKKYQDLKKQTEVD-------DLEGGQDGQ-P
Solyc10g008570   1 MN--------------------DLFSPSLKKYQDLKQQVQMD-------DLELGTGGTGP
Solyc10g081580   1 MN--------------------DLLNDDDNFDAPRHQSNRNG-------DVEMGIQIPMN
Solyc01g056810   1 M-----------------------------------------------------------
Solyc07g052470   1 MN--------------------DLLADS-SFIAGKDNASKES-------DIEMGNRFTRS
Solyc06g053760   1 MN--------------------DLMTKSFTSYIDLKKAAMKDVEASPD--LEMGM----T
Solyc02g085090   1 MN--------------------DLMTKSFLSYMELKKQAHLDLET--ERDLEMGQ-----
Solyc01g109750   1 M-------------------------TKSFLSYVELKKQAMMDVEA--GPDIEMGQ-----
Solyc06g062360   1 MSFQD---------------------------------------------LESGRS---V
Solyc11g066910   1 MSFQD---------------------------------------------LDSGRS---S
Solyc08g005200   1 MSFQD---------------------------------------------LESGRP---L
Solyc08g076540   1 MSFQD---------------------------------------------LEAGRP---L
AtSYP22          1 MSFQD---------------------------------------------LESGRG---R
AtSYP23          1 MSFQD---------------------------------------------LEAGRGRSLA
AtSYP21          1 MSFQD---------------------------------------------LEAGT----R
Solyc06g072760   1 MSFED---------------------------------------------LESGSSLYVQ
AtSYP41          1 MATRNRTLLFRKYRNSLRSVRAPLSSSS---------LTGTRSG-GVGPVIEMASTSLLN
AtSYP43          1 MATRNRTLLFRKYRNSLRSVRAPMGSSSSSTLTEHNSLTGAKSG--LGPVIEMASTSLLN
Solyc01g100170   1 MASRNRTLVFRKYRDALRSVRIPAGSS-----------TSTSSGHGSGPVIELATTSLLN
AtSYP42          1 MATRNRTTVYRKHRDACKSARAPLSLSA--------------SDSFGGPVIEMVSGSFSR
Solyc09g075530   1 MATRNRIRVFQSYRDTLNENRIPFTTSKDS----------------GGPVIEMATTSFLN
Solyc03g033850   1 M-----------------------------------------------------------
Solyc04g071730   1 MS----------------------------------------------------------
AtSYP81          1 MS----------------------------------------------------------
AtSYP31          1 M-GS----TFRDRTVELH---------------------------------SLSQTLKKI
Solyc07g054140   1 M-ASSGAWTYRDRTSEFA---------------------------------SLSKTLKKI
AtSYP32          1 MSARHGQSSYRDRSDEFF---------------------------------KIVETLRRS
Solyc08g067910   1 MPVKVASASLRDRTQEFQ---------------------------------SIAERLKKS
```

Ha

```
AtPEN1          40 G----GVNLDKFFEDVESVKEELKE--------------------LDRLNETLSSCHEQS
VvPEN1          28 A----GVNLDKFFEDVESIKEELRE--------------------MESLQQKLHDAHEQS
HvROR2          37 A----GASLDRFFEDVESIKDDLRE--------------------LERIQRSLHDGNESG
SlPEN1a         33 G----GVNLDKFFEDVETIKDELKE--------------------LEKIHTQLHNSHEQS
SlPEN1b         30 G----GVNLDKFFEDVEAIKDELKN--------------------LEKIYAQLQSSNEKS
AtSYP122        35 GSCHGGNNLDTFFLDVEVVNEDLKE--------------------LDRLCHNLRSSNEQS
AtSYP124        29 ---E-TMNLDKFFEDVENVKDNMKG--------------------VETLYKSLQDSNEEC
AtSYP123        32 ---D-SGNLDEFFGYVESVKEDMKA--------------------VDEIHKRLQDANEES
AtSYP111        37 KADKMDENLSSFLEEAEYVKAEMGL--------------------ISETLARIEQYHEES
AtSYP131        30 SG---DLGLSGFFKKVQEIEKQYEK--------------------LDKHLNKLQGAHEET
AtSYP125        24 ---E-TMNLDKFFEDVENVKDDMKG--------------------VEALYKKLQDSNEEC
AtSYP132        29 GG---DQGLEDFFKKVQVIDKQYDK--------------------LDKLLKKLQASHEES
AtSYP112        39 FSPADEENLSGFFQEIETIKTLIEE--------------------ITHLLLDLQNLNEET
Solyc12g005580  33 G-TE-SIDLAKFFEDVENVKEDMKD--------------------VEKFHKKLQESNEES
Solyc10g008570  34 SHNE-SIDLAKFFEDVENVKEDMKE--------------------VEKLHKRLQDSNEES
Solyc10g081580  34 SG---ELGLDDFFKKVQQIEKQYGR--------------------LNELLQKLQDAHEES
Solyc01g056810   2 ------------------------------------------------LALSLIDAHEES
Solyc07g052470  33 QS---DSGIDSFNKQIQEIEKQVDR--------------------LSGLLKTLKDANEET
Solyc06g053760  35 Q---MDQNLTAFLEEAEKVKLEMNS--------------------IKEILRRLQDTNEES
Solyc02g085090  34 LSRTDEDNLSNFFREIEAVKGDIQE--------------------ITNLLMDLQNLNEET
Solyc01g109750  30 LDPTDERNLSKFFEEVAVIKSDMEE--------------------INNLLVNLQDLNRKT
Solyc06g062360  13 GPRRGFM-------------NG-KQ----------------------------DTTQAVA
Solyc11g066910  13 GPRRSNV-------------NG-KQ----------------------------DATQALA
Solyc08g005200  13 GSRRFQT-------------NG-KQ----------------------------DPTQAVA
Solyc08g076540  13 GPRRGYM-------------NG-KQ----------------------------DPTQAVA
AtSYP22         13 STRKFN--------------GG-RQ----------------------------DSTQAVA
AtSYP23         16 SSRNING-------------GGSRQ----------------------------DTTQDVA
AtSYP21         12 SPAPNRF-------------TGGRQ--------------------QRPS-SRGDPSQEVA
Solyc06g072760  16 GGSRSWE-------------RQTTQ--------------------AITNPSASDNRQSIV
AtSYP41         51 PNRS-YAPISTEDP-GTS-SKGAIT--------------------VGLPPAWVDVSEEIS
AtSYP43         59 PNRS-YAPVSTEDP-GNS-SRGTIT--------------------VGLPPDWVDVSEEIS
Solyc01g100170  50 PNRS-YAPLSTEDP-GTS-SNGPVT--------------------VGLPPAWVDLSDEIT
AtSYP42         47 SNHSSYAPLNSYDP-GPS-SSDAFT--------------------IGMPPAWVDDSEEIT
Solyc09g075530  45 SNRS-YAPLSTEDDLGPSTSRDAFT--------------------LGLPPAWVDVSEEVA
Solyc03g033850   2 ----------------------------------------------HKPRQRLGFTRAAI
Solyc04g071730   3 ----------KVRDRTEDFKDVAHRSALSLGYDESKTAALLASFIMHKPRQKSGFTRAAL
AtSYP81          3 ----------RFRDRTEDFKDSVRNSAVSIGYNESKVASTMASFIIHKPKERSPFTKAAF
AtSYP31         23 -GAIPSVHQ-----DEDDPASSKRS----SPGSEFNKKASRIGLGIKETSQKITRLAK--
Solyc07g054140  27 AGTTGSDHE-----PQQNSASSTTKVLQIPDRSEFNKKASRIGLTIHQTFQKIDRLAK--
AtSYP32         28 IAPAPAANNVPYGNNRNDGARRED----LINKSEFNKRASHIGLAINQTSQKLSKLAKRI
Solyc08g067910  28 FS---SVQNGSISTSTSSGSRSEEQRTTIAMQSEFNRRASKIGFGIHQTSQKLAKLAK--
```

Hb

```
AtPEN1          76 KTLHNAKAVKD--LRSKMD-----------GDVGV--ALKKAKMIKVKLEAL------DR
VvPEN1          64 KTLHNANSVKE--LRSRMD-----------SHVSL--ALKKAKLIKLRLEAL------DR
HvROR2          73 KSLHDASAVRA--LRSRMD-----------ADVAA--AIKKAKVVKLRLESL------DR
SlPEN1a         69 KTLHNAKNVKD--LRKKMD-----------NDVSL--ALKKAKFIKVRLEAL------DR
SlPEN1b         66 KTLHNAKAVKD--LRSKMD-----------DDVSL--ALKKAKFIKVRLEAL------DR
AtSYP122        75 KTLHNANAVKE--LKKKMD-----------ADVTA--ALKTARRLKGNLEAL------DR
AtSYP124        65 KTVHNAKKVKE--LRAKMD-----------GDVAQ--VLKRVKMIKQKLEAL------EK
AtSYP123        68 KTVHDSKAVKK--LRARMD-----------SSVTE--VLKRVKMIKTKLVAL------EK
AtSYP111        77 KGVHKAESVKS--LRNKIS-----------NEIVS--GLRKAKSIKSKLEEM------DK
AtSYP131        67 KAVTKAPAMKS--IKQRME-----------RDVDE--VGRISRFIKGKIEEL------DR
AtSYP125        60 KTVHNAKKVKE--LRAKMD-----------GDVAM--VLKRVKIIKQKLEAL------EK
AtSYP132        66 KSVTKAPAMKA--IKKTME-----------KDVDE--VGSIARFIKGKLEEL------DR
AtSYP112        79 KSTHSTKILRG--LRDRME-----------SNIVT--ISRKANTVKTLIETL------EK
Solyc12g005580  71 KLVHNAKTVKE--IRSRMD-----------SDVSQ--VLKRVKMIKGKLEAL------ER
Solyc10g008570  73 KTVHSAKKVKD--IRARMD-----------SDVTL--VLKRVKIIKGKLEGL------ER
Solyc10g081580  71 KAVTKASAMKA--IKQRME-----------KDVDE--VGKIARVIKSKIEEL------DK
Solyc01g056810  14 KAVTKATAMKA--IKQRME-----------KDVDE--VSKVARFIKSKIEGL------DK
Solyc07g052470  70 KSVTKASAMKA--IRKRME-----------KDIDE--VGKIARNVKAKIEAT------NK
Solyc06g053760  72 KSLHKPEALKS--MRDRIN-----------SDIVA--VLKKARAIRSQLEEM------DR
Solyc02g085090  74 KTTHGPKVLRG--IRDRMD-----------SDMVS--VLRKAKIVKAKLEAL------DK
Solyc01g109750  70 KSAPSAKILQG--HRDQIN-----------SDIIT--VLRKAKMIKTRLELL------DK
Solyc06g062360  31 SGIFQINTAVS--TFQR-----LVNTLGTPKDTPE--LRDKLHKTRLHIGQL------VK
Solyc11g066910  31 SGIFQINTAVS--TFQR-----LVNTLGTPKDTPE--LRDKLHKTRLHIGQL------VK
Solyc08g005200  31 SGIFQINTAVS--TFQR-----LVNTLGTPKDTPE--LRDKLHKTRVHIGQL------VK
Solyc08g076540  31 SGIFQINTAVS--TFQR-----LVNTLGTPKDTPE--LREKLHKTRVHIGQL------VK
AtSYP22         30 SGIFQINTGVS--TFQR-----LVNTLGTPKDTPE--LREKLHKTRLHIGQL------VK
AtSYP23         35 SGIFQINTSVS--TFHR-----LVNTLGTPKDTPE--LREKLHKTRLYIGQL------VK
AtSYP21         38 AGIFRISTAVN--SFFR-----LVNSIGTPKDTLE--LRDKLQKTRLQISEL------VK
Solyc06g072760  43 VGVFQINTALT--NFQR-----LVNTLGTPKDTLQ--LRHKLHSTRQQIAEL------IK
AtSYP41         88 VNIQRARTKMA--ELGKAHAKALMPSFG---DGKE--DQHNIESLTQEITFL------LK
AtSYP43         96 VYIQRARTKMA--ELGKAHAKALMPSFG---DGKE--DQHQIETLTQEVTFL------LK
Solyc01g100170  87 ANVHRVRTKMS--ELAKAHAKALMPSFG---DGKE--DQRRIEALTHEITDL------LK
AtSYP42         85 FNIQKVRDKMN--ELAKAHSKALMPTFG---DNKG--IHREVEMLTHEITDL------LR
Solyc09g075530  84 ASIHQAQVKLA--DLKKCHAKALTPSFG---DGRE--DQNVIEVLTMEITDI------LR
Solyc03g033850  16 KTLESIGTLEQFLMKHKKDYVDLHRTTEQERDSIEHEVTIFVKSCKEQIDVLRNSINEED
Solyc04g071730  53 KTLESIGTLEQFLMKHKKDYVDLHRTTEQERDSIEHEVTIFVKSCKEQIDVLRNSINEED
AtSYP81         53 KTLDSIKELELFMLKHRKDYVDLHRTTEQEKDSIEQEVAAFIKACKEQIDILINSIRNEE
AtSYP31         71 ------------LAKQS-----------TIFNDRTVEIQELTVLIRNDITGLNMALSDLQ
Solyc07g054140  80 ------------LAKRS-----------SIFDDPSKEIQELTTSIKNDITSLNVGVSDLQ
AtSYP32         84 RMVLRSRTDLFSVAKRT-----------SVFDDPTQEIQELTVVIKQEISALNSALVDLQ
Solyc08g067910  83 ------------LAKRT-----------SVFDDPTTEIQELTAVIKQDITALNSAVVDLQ
```

Hc

```
AtPEN1          115 ANAANRSLP-GCGPGSSSDRTRTSVLNGLRKKLMDSMDSFNRLRELISSE--YRETVQRR
VvPEN1          103 SNAANRSLP-GCGPGSSSDRTRTSVVNGLRKKLRDSMDAFTSIRNQISSE--YRETVQRR
HvROR2          112 ANAANRSVA-GCGPGSSTDRTRTSVVAGLRKKLRDAMESFSSLRSRITSE--YRETVARR
SlPEN1a         108 SNAANRSVP-GCGPGSSSDRTRTSVVNGLRKKLQESMNQFNELRQRMASE--YRETVQRR
SlPEN1b         105 SNASNRSLP-GCGPGSSSDRTRTSVVNGLRKKLQESMNQFNELRQKMASE--YRETVQRR
AtSYP122        114 ANEVNRSLP-ESGPGSSSDRQRTSVVNGLRKKLKDEMEKFSRVRETITNE--YKETVGRM
AtSYP124        104 ANANSRNVS-GCGPGSSTDRTRTSVVSGLGKKLKDLMDSFQGLRARMNAE--YKETVERR
AtSYP123        107 SNAAQRKVA-GCGPGSSADRTRTSVVSGLGKKLKDMMDDFQRLRTKMATE--YKETVERR
AtSYP111        116 ANKEIKRLS-G----TPVYRSRTAVTNGLRKKLKEVMMEFQGLRQKMMSE--YKETVERR
AtSYP131        106 ENLENRTKP-GCGKGTGVDRTRTATTIAVKKKFKDKISEFQTLRQNIQQE--YREVVERR
AtSYP125         99 ANANSRNVP-GCGPGSSTDRTRSSVVSGLGKKLKDLMDSFQGLRARMNNE--YKETVERR
AtSYP132        105 ENLANRQKP-GCAKGSGVDRSRTATTLSLKKKLKDKMAEFQVLRENIQQE--YRDVVDRR
AtSYP112        118 RNVANR---TSFKEGSCVDRTRTSITNGVRKKLRDTMSEFHRLRERIFAD--YREDLKRK
Solyc12g005580  110 SNAAHRKIS-GCGPGSSADRTRTSVVSGLGKKLKVLMDDFQGLRTRMNDE--YKETVARR
Solyc10g008570  112 SNVANRKNL-GCGPGSSADRTRTSVVSGLGKKLKVLMDDFQALRAKMNSE--YKDTVARR
Solyc10g081580  110 ENLANRNKP-GCGKGSAVDRSRTATTVSLKKKFKDKMAEFQTLRENIHHE--YREVVERR
Solyc01g056810   53 ENLSNRSKP-GCGKGSAVDRSRTATTVSLKKKLKDKMSEFQTLRENIHNE--YREVVERR
Solyc07g052470  109 ENLANLQKP-GCGKGTSVDRSRTNMTNSLTKKFRDVMTEFQTLRQRIDNE--YREVVERR
Solyc06g053760  111 SNAINRRLS-GCKEGTLVDRTRSAVTNGLRKKLKELMMDFQGLRQRMMTE--YKETVGRR
Solyc02g085090  113 SNVGNRKLSVAYAQGSVVDRTRVSMSNGLRVKLRDIMNDFQALREKILSD--YKDCLRRR
Solyc01g109750  109 SNLDNRGVS-----GSPVDRTRISVTNGLRIKLRDMMNDFQCLRENIVAE--HKEGLRKQ
Solyc06g062360   76 DTSTKLKQASETDHRVEVSASKKITDAKLAKDFQAVLKEFQKAQRLAAER--ETAYTPFI
Solyc11g066910   76 DTSAKLKQASETDHRIEVSASKKITDAKLAKDFQAVLKEFQKAQRLAAER--ETSYSPFV
Solyc08g005200   76 DTSAKLKQASETDHHADVSASKKITDAKLAKDFQAVLKEFQKAQRLAAER--ETAYTPFI
Solyc08g076540   76 DTSAKLKQASETDHRVEVSASKKITDAKLAKDFQAVLKEFQKAQRLAAER--ETAYTPFV
AtSYP22          75 DTSAKLKEASETDHQSGVNPSKKIADAKLARDFQAVLKEFQKAQQTAAER--ETTYTPFV
AtSYP23          80 DTSAKLKEASETDHQRGVNQKKKIVDAKLAKDFQAVLKEFQKAQRLAAER--ETVYAPLV
AtSYP21          83 NTSAKLKEASEADLHGSASQIKKIADAKLAKDFQSVLKEFQKAQRLAAER--EITYTPVV
Solyc06g072760   88 ETSANLKQAIGSNRHSQSSVTKKIANAKLAKDFQSVLKKFQRAQQLAAQR--EAAFTPSI
AtSYP41         135 KSEKQLQRLSASGPSEDSNVRKNV-QRSLATDLQLLSMELRKKQSTYLKR----------
AtSYP43         143 KSEKQLQRLSAAGPSEDSNVRKNV-QRSLATDLQNLSMELRKKQSTYLKR----------
Solyc01g100170  134 RSEKKLQRLSAAGLSEDSNVRKNV-QRSLATDLQSLSMELRRKQSTYLKR----------
AtSYP42         132 KSEKRLQMLSTRGPSEESNLRKNV-QRSLATDLQNLSMELRRKQSTYLKR----------
Solyc09g075530  131 KSQKKLQKLSASGSCEDSNVRKNV-QRSLATDLQNLSVELRRMQSLYLKQ----------
Solyc03g033850   76 ANSKGWLGLKGDNLNADTIAHKHGVVLILSEKLHSVTSQFDQLRAIRFQDAINRVTPRRN
Solyc04g071730  113 ANSKGWLGLKGDNLNADTIAHKHGVVLILSEKLHSVSSQFDQLRAIRFQDAINRVTPRRK
AtSYP81         113 ANSKGWLGLPADNFNADSIAHKHGVVLILSEKLHSVTAQFDQLRATRFQDIINRAMPRRK
AtSYP31         108 TLQNMELADGNYSQ--DQVGHYTAVCDDLKTRLMGATKQLQDVLTTRSENMKAHENRKQL
Solyc07g054140  117 ALQDMDVADGTHSK--DTIVHCTAICDDLKTRLMAATKSFQEALTIRTKNMKAHEDRKQI
AtSYP32         133 LFRSSQNDEGNNSRDRDKSTHSATVVDDLKYRLMDTTKEFKDVLTMRTENMKVHESRRQL
Solyc08g067910  120 LHSNARNESGNS----DTTSHSTTVVDDLKNRLMTATKEFKEVLTMRTENMKVHENRRQM
```

```
AtPEN1          172 YFTVTGENPDERTL--------------------------------------------DR
VvPEN1          160 YFTVTGENPDEKTV--------------------------------------------DL
HvROR2          169 YFTVTGSQPDEATL--------------------------------------------DT
SlPEN1a         165 YFTVTGENPDEGTL--------------------------------------------DT
SlPEN1b         162 YYTVTGENPDEAVL--------------------------------------------DT
AtSYP122        171 CFTVTGEYPDEATL--------------------------------------------ER
AtSYP124        161 YFTITGEQADEQTI--------------------------------------------EN
AtSYP123        164 YFTVTGQKADEETV--------------------------------------------EK
AtSYP111        169 YFTVTGEHANDEMI--------------------------------------------EK
AtSYP131        163 VFTVTGQRADEEAI--------------------------------------------DR
AtSYP125        156 YFTITGEKADEQTI--------------------------------------------DN
AtSYP132        162 VYTVTGERADEDTI--------------------------------------------DE
AtSYP112        173 YFLATGEEPSNEDM--------------------------------------------EK
Solyc12g005580  167 YFTVTGEKADDGLI--------------------------------------------EN
Solyc10g008570  169 YFTVTGENADDELI--------------------------------------------DN
Solyc10g081580  167 VFTVTGNRADEETI--------------------------------------------DR
Solyc01g056810  110 VYTVTGNRADEETI--------------------------------------------DR
Solyc07g052470  166 VITVTGTRPDEETI--------------------------------------------NN
Solyc06g053760  168 YFTVTGEHPDEEVI--------------------------------------------DK
Solyc02g085090  171 YYNETGKEPNEEVI--------------------------------------------EK
Solyc01g109750  162 YSNANGKEPSEEAI--------------------------------------------EK
Solyc06g062360  134 PQAVLPSSYTDGEV--------------------------------------------DV
Solyc11g066910  134 PQAVLPSSYTNSEI--------------------------------------------DV
Solyc08g005200  134 PQAVLPSSYTASEV--------------------------------------------DV
Solyc08g076540  134 PQAVLPSSYTASEI--------------------------------------------DV
AtSYP22         133 PQSALPSSYTAGEV--------------------------------------------D-
AtSYP23         138 HKPSLPSSYTSSEI--------------------------------------------DV
AtSYP21         141 TKE-IPTSYNAPEL--------------------------------------------DT
Solyc06g072760  146 SQEI--NSSRSIEI--------------------------------------------QI
AtSYP41         184 ---LRQQKE--DGM--------------------------------------------DL
AtSYP43         192 ---LRLQKE--DGA--------------------------------------------DL
Solyc01g100170  183 ---LQQQKEGPDGV--------------------------------------------DL
AtSYP42         181 ---LQQQKEGQDEV--------------------------------------------DL
Solyc09g075530  180 ---LRLQSEGHDGL--------------------------------------------DL
Solyc03g033850  136 RKSTTKSNAAEASA--------------------------------------------SI
Solyc04g071730  173 RKNTTKSNAAETSV--------------------------------------------SS
AtSYP81         173 PKRVIKE---ATPI--------------------------------------------NT
AtSYP31         166 FSTKNAVD--SP--------PQNNAKSVPEPPPWSSSSNPFGNLQQPLLPPLNTGAPPGS
Solyc07g054140  175 FSTNLSRE--NP--------LK---QPTAEPPPWSTCQS---------LTAID--AQGSN
AtSYP32         193 FSSNASKESTNPFVRQRPLAAKAAASESV-PLPWANGSS-----------------SSSS
Solyc08g067910  176 FSSSTSKEASNPFMRQRPLASRNTASTSASPPPWAN-DS-----------------PSSS
```

```
AtPEN1          188 LIS---TGES---ERFLQKAIQEQ-GRGRVLDTINEI----------QERHDAVKDIEKN
VvPEN1          176 LIS---TGES---ETFLQKAIQEQ-GRGRVLDTISEI----------RERHESVKELERN
HvROR2          185 LAE---TGEG---ERLLQRAIAEQQGRGEVLGVVAEI----------QERHGAVADLERS
SlPEN1a         181 LIS---TGQS---ETFLQKAIQEQ-GRGQVMDTVMEI----------QERHEAVKELERN
SlPEN1b         178 LIS---TGQS---ETFLQKAIQEQ-GRGQVMDTIMEI----------QERHEAVKEIERN
AtSYP122        187 LIS---TGES---ETFLQKAIQEQ-GRGRILDTINEI----------QERHDAVKDIEKS
AtSYP124        177 LIS---SGES---ENFLQKAIQEQ-GRGQILDTISEI----------QERHDAVKEIEKN
AtSYP123        180 LIS---SGES---ERFLQKAIQEQ-GRGQVMDTLSEI----------QERHDTVKEIERS
AtSYP111        185 IIT-----DNAGGEEFLTRAIQEH-GKGKVLETVVEI----------QDRYDAAKEIEKS
AtSYP131        179 LIE---TGDS---EQIFQKAIREQ-GRGQIMDTLAEI----------QERHDAVRDLEKK
AtSYP125        172 LIA---SGES---ENFLQKAIQEQ-GRGQILDTISEI----------QERHDAVKEIEKN
AtSYP132        178 LIE---TGNS---EQIFQKAIQEQ-GRGQVMDTLAEI----------QERHDAVRDLEKK
AtSYP112        189 MIS---GSGS-------------CSDLVKTFEVKPEM------DLKTKERHEAVNDIKRS
Solyc12g005580  183 LIS---SGES---ESFLQKAIQEQ-GRGQIMDTISEI----------QERHDAVKEIEKN
Solyc10g008570  185 LIS---SGES---ESFLQKAIQEQ-GRGQIMDTISEI----------QERHDAVKEIEKN
Solyc10g081580  183 LIE---TGDS---EQIFQKAIQQQ-GRGQIMGTLAEI----------QERHDAVRELERK
Solyc01g056810  126 LIE---TGDS---EQIFQKAIREQ-GRGQIMDTLAEI----------QERHDAVRELERK
Solyc07g052470  182 LIE---TGNS---EQIFQNAIQGM-GRGQVLSTVEEI----------QERHDAVKEIERK
Solyc06g053760  184 IIS---SGNGQGGEEFLSRAIQEH-GRGKVLETVVEI----------QDRHDAAKEIEKS
Solyc02g085090  187 MVS---GE----------------SGKVQIFAAKTEM------NLDDKDRHEAVMDIKKS
Solyc01g109750  178 MMQ---ERVI-------------EKGVVE-----------------NQDRHEAVKEIQKS
Solyc06g062360  150 SSD---KGQE-------QRALLVESRRQEVLLLDNEISF---NEAIIEEREQGIQEVQQQ
Solyc11g066910  150 SSD---KSQE-------QRALLVESRRQEVVLLDNEISF---NEAIIEERDQGIQEVQQQ
Solyc08g005200  150 ASD---KSQE-------QRALLVESRRQDVLYLDNEIAF---NEAIIEERDLGIQEVQQQ
Solyc08g076540  150 SSG---KSPE-------QRALLVESRRQEVLLLDNEIAF---NEAIIEEREQGIQEIQQQ
AtSYP22         148 ------KVPE-------QRAQLQESKRQELVLLDNEIAF---NEAVIEEREQGIQEIHQQ
AtSYP23         154 NGD---KHPE-------QRALLVESKRQELVLLDNEIAF---NEAVIEEREQGIQEIQQQ
AtSYP21         156 ESL---RISQ-------QQALLLQSRRQEVVFLDNEITF---NEAIIEEREQGIREIEDQ
Solyc06g072760  160 SSS---ISPE-------SSSILLESKRQDVVQLEHEIVF---NKAIIEEREQGMIEIQQQ
AtSYP41         195 EMN---LSRN-------RYR-PEEDDFGDML-NEHQMSKIKKSEEVSVEREKEIQQVVES
AtSYP43         203 EMN---LNGS-------RYK-AEDDDFDDMVFSEHQMSKIKKSEEISIEREKEIQQVVES
Solyc01g100170  196 EMN---LNGS-------HSR-RDDDDLDDLGFNEHQMAKLKKSEAFTVEREREIQQVVES
AtSYP42         194 EFN---VNGK-------MSRLDEEDELGGMGFDEHQTIKLKEGQHVSAEREREIQQVLGS
Solyc09g075530  193 EMN---EKKS-------SFL---DDDFNDVGFTELQMATGQKDEQFTAEREREIRQVLKS
Solyc03g033850  152 SLDPDMKRDSEVRDNDVSQAAPMRVQEQ-LLDDETRALQVELNSLL-----DSVQETETN
Solyc04g071730  189 NLDPNMKRDSEGLGDPDTQAAPIRVQEQ-LLDDETRALQVELNSLL-----DSVQETETK
AtSYP81         186 TLG-----NSESIEPDEIQAQPRRLQQQQLLDDETQALQVELSNLL-----DGARQTETK
AtSYP31         216 QL--RRRSAIENAP----SQQMEMSLLQQTVPKQENYS---------QSRAVALHSVESR
Solyc07g054140  211 QL--RRRLASDNPP----SNELEMSMLQDQVPRQESYS---------QSRATALQNVEST
AtSYP32         235 QLVPWKPGEGESSPLLQQSQQQQQQQQQQMVPLQDTYM---------QGRAEALHTVEST
Solyc08g067910  218 QLFPRKQGDGDTQPLLQD---QQQQQQQQIVPLQDSYM---------QSRAEALQNVEST
```

Qa- SNARE

```
AtPEN1          231 LRELHQVFLDMAVLVEHQGAQLDDIESHVGRASSFIRGGTDQLQTARVYQKNTR------
VvPEN1          219 LKELHQVFLDMAVLVQAQGEQLDDIESQVARANSFVTGGTQQLQTARKHQISSR------
HvROR2          229 LLELQQVFNDMAVLVAAQGEQLDDIEGHVGRARSFVDRGREQLQVARKHQKSSR------
SlPEN1a         224 LKELHQVFMDMAVLVESQGAQLDDIESQVNRANSFVRGGAQQLEVARKHQKSSR------
SlPEN1b         221 LKELHQVFLDMAVLVESQGEQLDDIESQVNRANSFVRGGAQQLQVARKHQKNTR------
AtSYP122        230 LNELHQVFLDMAVLVEHQGAQLDDIEGNVKRANSLVRSGADRLVKARFYQKNTR------
AtSYP124        220 LIELHQVFLDMAALVESQGQQLNDIESHVSKASSFVRRGTDQLQDAREYQKSSR------
AtSYP123        223 LLELHQVFLDMAALVEAQGNMLNDIESNVSKASSFVMRGTDQLHGAKVLQRNNR------
AtSYP111        229 LLELHQVFLDMAVMVESQGEQMDEIEHHVINASHYVADGANELKTAKSHQRNSR------
AtSYP131        222 LLDLQQVFLDMAVLVDAQGEMLDNIENMVSSAVDHVQSGNNQLTKAVKSQKSSR------
AtSYP125        215 LLELHQVFLDMAALVEAQGQQLNNIESHVAKASSFVRRGTDQLQDAREYQKSSR------
AtSYP132        221 LLDLQQIFLDMAVLVDAQGEMLDNIESQVSSAVDHVQSGNTALQRAKSLQKNSR------
AtSYP112        227 LNRLHQVFLDMAVLVETQGDRIDDIEANVANAGSFVSGGTNSLYYANQMKKKTK------
Solyc12g005580  226 LIELHQIFLDMAALVEAQGQQLNDIESHVAHASSFVRRGTEQLQEAREIQKSSR------
Solyc10g008570  228 LIELHQIFLDMAALVEAQGQQLNDIESHVAHASSFVRRGTEQLTEARELQKSSR------
Solyc10g081580  226 LLELQQIFLDIAVLVDAQGDMLDNIESQVSTAVDHVQSGTTALQKAKKLQKNSR------
Solyc01g056810  169 LLELQQIFLDMAVLVDAQGDMLDNIESQVSAAVDHVQSGNTALQKAKSLQRNSR------
Solyc07g052470  225 LLDLHQIYLDMAVLVEAQGDLLDNIETQVRYAVDHVNMGTDALQTAKSLQKKSR------
Solyc06g053760  230 LLELHQIFLDMAVMVEAQGEKMDDIEHHVVNAAQYVNDGAKNLKTAKKYQKSSR------
Solyc02g085090  222 LDKLHQVFLDMAVLVETQGEQIDDIEHNMAIAGSFISGGTNSLFYAKQQQKKGR------
Solyc01g109750  205 LVELHQVFLDMAVMVETQGDQMNNIEQNVVNAGGYVNGGMKELDRANRM-KRTR------
Solyc06g062360  197 IGEVNEIFKDLAVLVHEQGTMIDDIGSNIENSHAATAQGRSQLAKAAKTQRSNS------
Solyc11g066910  197 IGEVNDIFKDLAVLVHEQGTMIDDIGSNIENSHAATAQGRTQLAKAAKTQRSNS------
Solyc08g005200  197 IGEVNEIFKDLAVLVHEQGTMIDDIGSNIENSHAATALGRSQLAKAAKTQRSNS------
Solyc08g076540  197 IGEVNEIFKDLAVLVHEQGAMIDDIGSNVENAHAATAQGRSQLAKAAKTQRSNS------
AtSYP22         192 IGEVNEIFKDLAVLVNDQGVMIDDIGTHIDNSRAATSQGKSQLVQAAKTQKSNS------
AtSYP23         201 IGEVHEIFKDLAVLVHDQGNMIDDIGTHIDNSYAATAQGKSHLV---RHQRHKD------
AtSYP21         203 IRDVNGMFKDLALMVNHQGNIVDDISSNLDNSHAATTQATVQLRKAAKTQRSNS------
Solyc06g072760  207 IGELNEMFKDLALLVHEQGTMLDDISSNIGSSHDATAQAAKQLTKASKIQQCNS------
AtSYP41         243 VNDLAQIMKDLSALVIDQGTIVDRIDYNIENVATTVEDGLKQLQKAERTQRHGG------
AtSYP43         252 VSELAQIMKDLSALVIDQGTIVDRIDYNIQNVASTVDDGLKQLQKAERTQRQGG------
Solyc01g100170  245 VNDLAQIMKDLSVLVIDQGTIVDRIDHNIQNVASTVEDGLKQLKKAERSQKRGG------
AtSYP42         244 VNDLAQIMKDLSALVIDQGTIVDRIDYNVQNVSTSVEEGYKQLQKAERTQREGA------
Solyc09g075530  240 VNELAQIMKDLSVLVIDQGTIVDRIDHNVQSVSASVEEGFKQLQKAERSQRKGG------
Solyc03g033850  206 MVEMSALNHLMSTHVLQQAQQIELLYEQAVEATQNVELGNKELSQAIQRNSSSR------
Solyc04g071730  243 MVEMSALNHLMSTHVLQQAQQIELLYEQAVEATQNVELGNKELSQAIQRNSSSR------
AtSYP81         236 MVEMSALNHLMATHVLQQAQQIEFLYDQAVEATKNVELGNKELSQAIQRNSSSR------
AtSYP31         261 ITELSGIFPQLATMVTQQGELAIRIDDNMDESLVNVEGARSALLQHLTRISSNRWLMMKI
Solyc07g054140  256 ISELGGIFTHLATMVAQQGELAIRIDDNVDESLTNVEGAQGALLKYLNRISSNS---RKV
AtSYP32         286 IHELSSIFTQLATMVSQQGEIAIRIDQNMEDTLANVEGAQSQLARYLNSISSNRWLMMKI
Solyc08g067910  266 IHELGSIFNQLATLVSQQGEVAIRIDENMDDTLTNVEGAQGALLKYLNSISSNRW
```

```
AtPEN1          285 ------------------------------------------------------KWTCIA
VvPEN1          273 ------------------------------------------------------KWTCYG
HvROR2          283 ------------------------------------------------------KWTFIG
SlPEN1a         278 ------------------------------------------------------KWTCIA
SlPEN1b         275 ------------------------------------------------------KWTCFA
AtSYP122        284 ------------------------------------------------------KWTCFA
AtSYP124        274 ------------------------------------------------------KWTCYA
AtSYP123        277 ------------------------------------------------------KWACIA
AtSYP111        283 ------------------------------------------------------KWMCIG
AtSYP131        276 ------------------------------------------------------KWMCIA
AtSYP125        269 ------------------------------------------------------KWTCYA
AtSYP132        275 ------------------------------------------------------KWMCIA
AtSYP112        281 ------------------------------------------------------SWVLWV
Solyc12g005580  280 ------------------------------------------------------KCACFA
Solyc10g008570  282 ------------------------------------------------------KCTCIA
Solyc10g081580  280 ------------------------------------------------------KWMCFA
Solyc01g056810  223 ------------------------------------------------------KWMCIA
Solyc07g052470  279 ------------------------------------------------------KCMMIA
Solyc06g053760  284 ------------------------------------------------------RCMCIG
Solyc02g085090  276 ------------------------------------------------------AWICWV
Solyc01g109750  258 ------------------------------------------------------TWACWI
Solyc06g062360  251 ------------------------------------------------------SLTC--
Solyc11g066910  251 ------------------------------------------------------SLTC--
Solyc08g005200  251 ------------------------------------------------------SLTC--
Solyc08g076540  251 ------------------------------------------------------SLTC--
AtSYP22         246 ------------------------------------------------------SLTC--
AtSYP23         252 ------------------------------------------------------QI----
AtSYP21         257 ------------------------------------------------------SLTC--
Solyc06g072760  261 ------------------------------------------------------STSC--
AtSYP41         297 ------------------------------------------------------MVKCAS
AtSYP43         306 ------------------------------------------------------MVMCAS
Solyc01g100170  299 ------------------------------------------------------MVMCAT
AtSYP42         298 ------------------------------------------------------MVKCAT
Solyc09g075530  294 ------------------------------------------------------MVKCAT
Solyc03g033850  260 ------------------------------------------------------TF----
Solyc04g071730  297 ------------------------------------------------------TF----
AtSYP81         290 ------------------------------------------------------TF----
AtSYP31         321 F-----------------------------------------------------------
Solyc07g054140  313 SCTENYTTAACTTCCTGCAAAGTTCATGAATAATATTCACTTTCTCACACCATACAGCGA
AtSYP32         346 F-----------------------------------------------------------
Solyc08g067910
```

MS

```
AtPEN1          291 IIILIIIITVVVLAVLKPWNNSSGGGG---------------------------------
VvPEN1          279 IIILIVIILLIVLFTVRPWENN--------------------------------------
HvROR2          289 IGILLVVILIIVIPIVLKNTNKSNNNNSQQ------------------------------
SlPEN1a         284 IIILLIIVLVVVL-SIQPWKK---------------------------------------
SlPEN1b         281 IILLLIIILIVVL-SIQPWKK---------------------------------------
AtSYP122        290 ILLLLIIVVLIVVFTVKPWESNGGGGG---------------------------------
AtSYP124        280 ILLFIVVFALLLI-----------------------------------------------
AtSYP123        283 TILAIVVVIVILF-----------------------------------------------
AtSYP111        289 IIVLLLIILIVVIPIITSFSSS--------------------------------------
AtSYP131        282 ILILLIIIIITVISVLKPWTQKNG------------------------------------
AtSYP125        275 IILFIVIFILLLI-----------------------------------------------
AtSYP132        281 IIILLIVVAVIVVGVLKPW-KNKS------------------------------------
AtSYP112        287 SILGVLILLVCVISML--------------------------------------------
Solyc12g005580  286 VFLIILLIILL--------------------TF---------------------------
Solyc10g008570  288 ILLIILLIIKLHLQIVSMFLKLLGHRHYSSVTFFTNPPSKESEIIHLCRSDLLSQAIKLL
Solyc10g081580  286 IMILLIIVAIIVVGVLKPWQSNKG------------------------------------
Solyc01g056810  229 IIILLIIVAVIVVGVLKPWNSNKG------------------------------------
Solyc07g052470  285 IILLLIIAAIIVLSVIKPWKK---------------------------------------
Solyc06g053760  290 AIILLILILVVIIPIATSFTKS--------------------------------------
Solyc02g085090  282 WAVLLIILVVCLIATL--------------------------------------------
Solyc01g109750  264 GALVLVFLLICLIAIL--------------------------------------------
Solyc06g062360  255 -LLLVIFGIVLLIVII--------------------------------------------
Solyc11g066910  255 -LLLVIFGIVLLIVII--------------------------------------------
Solyc08g005200  255 -LLLVIFGIILLIVII--------------------------------------------
Solyc08g076540  255 -LLLVIFGIVLLIVIV--------------------------------------------
AtSYP22         250 -LLLVIFGIVLLIVII--------------------------------------------
AtSYP23         254 -----------LLCLI--------------------------------------------
AtSYP21         261 -LLILIFGIVLLIVII--------------------------------------------
Solyc06g072760  265 -LLLVIFGVILLIIIV--------------------------------------------
AtSYP41         303 VLVILCFIMLLLLILK--------------------------------------------
AtSYP43         312 VLVILCFIMLVLLILK--------------------------------------------
Solyc01g100170  305 VLVIMCFIMLVLLILK--------------------------------------------
AtSYP42         304 ILLVLCLIMIVLLILK--------------------------------------------
Solyc09g075530  300 ILVIMCFVMLVLLVLK--------------------------------------------
Solyc03g033850  262 -LLLFLVVLTFSILFLDWYS----------------------------------------
Solyc04g071730  299 -LLLFLFVLTFSILFLDWYS----------------------------------------
AtSYP81         292 -LLLFFFVLTFSVLFLDWYS----------------------------------------
AtSYP31         322 -AVIILFLIVFLFFVA--------------------------------------------
Solyc07g054140  373 GACTACGATGAAGATA--------------------------------------------
AtSYP32         347 -FVLIAFLMIFLFFVA--------------------------------------------
Solyc08g067910
```

```
AtPEN1                ------------------------------------------------------------
VvPEN1                ------------------------------------------------------------
HvROR2                ------------------------------------------------------------
SlPEN1a               ------------------------------------------------------------
SlPEN1b               ------------------------------------------------------------
AtSYP122              ------------------------------------------------------------
AtSYP124          293 ---------PAL------------------------------------------------
AtSYP123          296 ---------PILFNTLLRP-----------------------------------------
AtSYP111              ------------------------------------------------------------
AtSYP131              ------------------------------------------------------------
AtSYP125          288 ---------PLL------------------------------------------------
AtSYP132              ------------------------------------------------------------
AtSYP112              ------------------------------------------------------------
Solyc12g005580    299 ---------PL-------------------------------------------------
Solyc10g008570    348 KSTEKISSKPIVYATLIQTCTKSHSFNHGVQFHTHVIKTGIETDRFVGNSLLALYFKLGS
Solyc10g081580        ------------------------------------------------------------
Solyc01g056810        ------------------------------------------------------------
Solyc07g052470        ------------------------------------------------------------
Solyc06g053760        ------------------------------------------------------------
Solyc02g085090        ------------------------------------------------------------
Solyc01g109750        ------------------------------------------------------------
Solyc06g062360        ------------------------------------------------------------
Solyc11g066910        ------------------------------------------------------------
Solyc08g005200        ------------------------------------------------------------
Solyc08g076540        ------------------------------------------------------------
AtSYP22               ------------------------------------------------------------
AtSYP23               ------------------------------------------------------------
AtSYP21               ------------------------------------------------------------
Solyc06g072760        ------------------------------------------------------------
AtSYP41               ------------------------------------------------------------
AtSYP43               ------------------------------------------------------------
Solyc01g100170        ------------------------------------------------------------
AtSYP42               ------------------------------------------------------------
Solyc09g075530        ------------------------------------------------------------
Solyc03g033850        ------------------------------------------------------------
Solyc04g071730        ------------------------------------------------------------
AtSYP81               ------------------------------------------------------------
AtSYP31               ------------------------------------------------------------
Solyc07g054140        ------------------------------------------------------------
AtSYP32               ------------------------------------------------------------
Solyc08g067910
```

```
AtPEN1                 ------------------------------------------------------------
VvPEN1                 ------------------------------------------------------------
HvROR2                 ------------------------------------------------------------
SlPEN1a                ------------------------------------------------------------
SlPEN1b                ------------------------------------------------------------
AtSYP122               ------------------------------------------------------------
AtSYP124               ------------------------------------------------------------
AtSYP123               ------------------------------------------------------------
AtSYP111               ------------------------------------------------------------
AtSYP131               ------------------------------------------------------------
AtSYP125               ------------------------------------------------------------
AtSYP132               ------------------------------------------------------------
AtSYP112               ------------------------------------------------------------
Solyc12g005580  301    -------------------W----------------------------------------
Solyc10g008570  408    NFLETRRFFDGMVYKDVVAWSSMITGYVRIGKPKISLELYGEMIDLGFEPNGFTLSAVIK
Solyc10g081580         ------------------------------------------------------------
Solyc01g056810         ------------------------------------------------------------
Solyc07g052470         ------------------------------------------------------------
Solyc06g053760         ------------------------------------------------------------
Solyc02g085090         ------------------------------------------------------------
Solyc01g109750         ------------------------------------------------------------
Solyc06g062360         ------------------------------------------------------------
Solyc11g066910         ------------------------------------------------------------
Solyc08g005200         ------------------------------------------------------------
Solyc08g076540         ------------------------------------------------------------
AtSYP22                ------------------------------------------------------------
AtSYP23                ------------------------------------------------------------
AtSYP21                ------------------------------------------------------------
Solyc06g072760         ------------------------------------------------------------
AtSYP41                ------------------------------------------------------------
AtSYP43                ------------------------------------------------------------
Solyc01g100170         ------------------------------------------------------------
AtSYP42                ------------------------------------------------------------
Solyc09g075530         ------------------------------------------------------------
Solyc03g033850         ------------------------------------------------------------
Solyc04g071730         ------------------------------------------------------------
AtSYP81                ------------------------------------------------------------
AtSYP31                ------------------------------------------------------------
Solyc07g054140         ------------------------------------------------------------
AtSYP32                ------------------------------------------------------------
Solyc08g067910
```

Fig. S1 Multialignment of plant syntaxins. The dataset includes all the Arabidopsis syntaxins (AtSYPs and AtPEN1), barley HvROR2, grapevine VvPEN1 and 21 putative syntaxins retrieved from the tomato proteome by this study (indicated by the SolGenomics Network database ID code). The black box includes the SYP1-clade syntaxins (in number of 21). The six syntaxins belonging to the SYP1b-subclade are highlighted in light red.

The position of characteristic domains of syntaxins (the three helix domain Ha, Hb and Hc, the Qa-SNARE domain and the membrane-spanning (MS) domain are indicated. Gray color indicates residues physio-chemically conserved throughout the dataset; green color indicates residues physio-chemically conserved in SYP1 syntaxins. Yellow color highlights 24 residues specifically conserved in syntaxins of the SYP1b subclade described in this study.

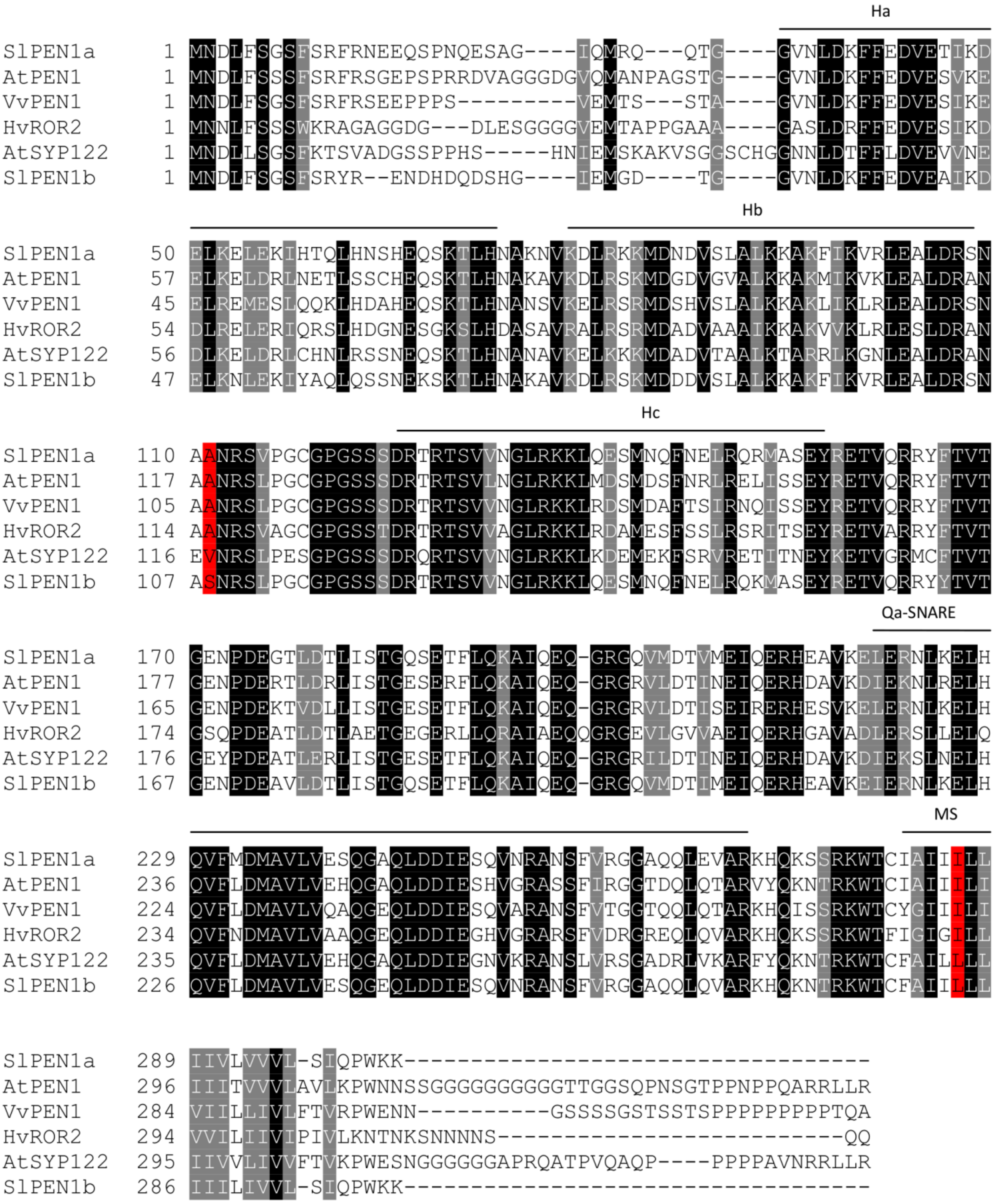

Fig. S2 Syntaxins of the SYP1b-subclade multialignment. The protein dataset includes Arabidopsis AtPEN1 and AtSYP122, tomato SlPEN1a and SlPEN1b, barley HvROR2 and grapevine VvPEN1.The position of characteristic syntaxin domains (the three helix domain Ha, Hb and Hc, the Qa-SNARE domain and the membrane-spanning (MS) domain are indicated. Red colour indicates amino acids differentially conserved in the syntaxins functionally characterized in plant-pathogen interaction.
